# Phenotypic and transcriptomic impact of expressing mammalian TET2 in the *Drosophila melanogaster* model

**DOI:** 10.1080/15592294.2023.2192375

**Published:** 2023-03-29

**Authors:** Joy N. Ismail, Sarah Mantash, Mohammad Hallal, Nada Jabado, Pierre Khoueiry, Margret Shirinian

**Affiliations:** a Department of Experimental pathology, Immunology and Microbiology, Faculty of Medicine, American University of Beirut, Beirut, Lebanon; b Department of Biochemistry and Molecular Genetics, Faculty of Medicine, American University of Beirut, Beirut, Lebanon; c Biomedical Engineering Program, American University of Beirut, Beirut, Lebanon; d Department of Human Genetics, McGill University, Montreal, Quebec, Canada; e Pillar Genomics Institute, Faculty of Medicine, American University of Beirut, Beirut, Lebanon

**Keywords:** TET2, *Drosophila melanogaster*, glioma, behaviour, circadian rhythm, innate immunity, leukaemia

## Abstract

Ten-Eleven Translocation (TET) proteins have recently come to light as important epigenetic regulators conserved in multicellular organisms. TET knockdown studies in rodents have highlighted the critical role of these proteins for proper brain development and function. Mutations in mammalian mTET proteins and mTET2 specifically are frequent and deregulated in leukaemia and glioma respectively. Accordingly, we examined the role of mTET2 in tumorigenesis in larval haemocytes and adult heads in *Drosophila melanogaster*. Our findings showed that expression of mutant and wild type mTET2 resulted in general phenotypic defects in adult flies and accumulation of abdominal melanotic masses. Notably, flies with mTET2-R43G mutation at the N-terminus of mTET2 exhibited locomotor and circadian behavioural deficits, as well as reduced lifespan. Flies with mTET2-R1261C mutation in the catalytic domain, a common mutation in acute myeloid leukaemia (AML), displayed alterations affecting haemocyte haemostasis. Using transcriptomic approach, we identified upregulated immune genes in fly heads that were not exclusive to TET2 mutants but also found in wild type mTET2 flies. Furthermore, inhibiting expression of genes that were found to be deregulated in mTET2 mutants, such as those involved in immune pathways, autophagy, and transcriptional regulation, led to a rescue in fly survival, behaviour, and hemocyte number. This study identifies the transcriptomic profile of wild type mTET2 versus mTET2 mutants (catalytic versus non-catalytic) with indications of TET2 role in normal central nervous system (CNS) function and innate immunity.

## Introduction

The ten-eleven translocation (TET) proteins are a family of epigenetic regulators discovered after identification of TET1 as a fusion partner of mixed lineage leukaemia (MLL) in acute myeloid leukaemia (AML) patients. This family of three proteins – TET1, TET2, and TET3 – plays a key role in active DNA demethylation [1]. TETs are dioxygenases that initiate active demethylation of cytosine residues on DNA [1–3]. They do so by catalysing the conversion of 5-methylcytosine (5mC) into 5hmC and further oxidized derivatives [4]. Enzymes involved in DNA repair can further remove these oxidized methylcytosines replacing the modified cytosines with unmodified ones. The catalytic activity of TET proteins is dependent on the presence of Fe (II) and 2-oxoglutarate as cofactors [5]. As TET proteins have an important role in regulating DNA methylation fidelity, their inactivation might contribute to DNA hyper-methylation of specific promoters often observed in cancer. TET2 mutations are commonly identified in myeloid and lymphoid malignancies and are amongst the most recurrently acquired mutations identified in patients suffering from chronic myelomonocytic leukaemia (CMML, 50–60%), AML (~20%), and myelodysplastic syndromes (MDS, ~20%) [6–8]. Furthermore, TET2 genetic alterations are observed in other types of haematological malignancies, including various B and T cell lymphomas [9]. TET1 and TET3 on the other hand are implicated in different cancers to a lesser extent – potentially due to their limited expression in adulthood relative to TET2. In a mouse model, TET1 deletion leads to B-cell lymphoma and a genetic dysregulation of DNA repair pathways [10]. While TET proteins were initially thought to be associated with blood cancers only, various recent studies have established that they may be involved in solid tumours also, further confirming their potential role in tumorigenesis. For instance, low levels of TETs 1–3 and 5hmC are observed in digestive system cancers, including gastric carcinoma and colorectal cancer [11–13]. TET1 is also found to be downregulated in breast, prostate, and lung cancers, associated with increased invasiveness and metastasis [14–17]. Due to the large number of additional somatic alterations found in many malignancies, it is difficult to conclude whether TET mutations are driving tumorigenesis or maintaining the tumorigenic status imposed by other oncogenes. Furthermore, it is not clear whether the catalytic activity or catalytic-independent mechanisms of TET are involved in oncogenesis. Therefore, a better understanding of the *in vivo* role of TET during oncogenesis is essential. In addition, the role of mutated TET proteins in solid cancers is not yet clearly established, although it is generally accepted that loss of TET function and low 5hmC levels are strongly associated with cancer [18]. Surprisingly, although the loss of function of TET proteins results in increased methylation at many regulatory genomic regions, a small number of studies have documented DNA hypomethylation in genomes of TET-deficient cells that were not found to overlap with active regulatory regions of the genome [19–21]. Therefore, this phenomenon of hypomethylation upon loss of TET function is poorly understood. In addition, there is increasing evidence from recent years that not only the loss of TET function is correlated with increased malignancy and poor prognosis, but the overexpression of TET proteins can also lead to unfavourable outcomes in cancer. For example, in a recent study TET3 upregulation was documented in hepatoblastoma with a concomitant increase in the 5hmC content in these tumour cells [22]. Furthermore, studies on animal models revealed that TET overexpression in embryonic developmental stages represses differentiation and alters cellular reprogramming of induced pluripotent stem cells (iPSCs) [23]. In this study, we opted to understand TET2 function in a simple, tractable *Drosophila* model. To this end, we generated transgenic flies expressing wild type mTET2, mTET2 with an Arginine to Glycine mutation (R43G) at the N-terminus identified in a diffuse intrinsic pontine glioma (DIPG) patient (found in a glioma cohort from our group), or mTET2 with an Arginine to Cysteine (R1261C) mutation in the catalytic domain that is frequently reported in AML. We investigated their impact on survival and behavioural phenotypes and their role in glial and haemocyte homoeostasis in the brain and haemolymph respectively. Our findings showed that expression of mutant and wild type TET2 resulted in general defects in the adult fly thorax and male genitalia, and accumulation of necrotic/melanotic masses in the abdomen. Notably, the R43G non-catalytic mutation conferred changes in behaviour and fly lifespan whereas the common leukaemia mutation R1261C had a stronger impact on larval haemocyte haemostasis. Through transcriptomic analysis, we further identified an immune signature that was consistently upregulated in all transgenic mTET2 fly heads, including the wild type mTET2, with a relatively small number of genes that were differentially expressed in R43G and R1261C compared to wild type. Remarkably, when some of these upregulated genes were knocked down in R43G and R1261C mutants, impairments in lifespan, behaviour, and haemocyte number defects were rescued suggesting that these genes could serve as potential candidates for further investigation in higher mammalian models or tissues from TET2 mutant tumour samples.

## Materials and methods

### Fly stocks and handling

The following fly stocks were obtained from the Bloomington Drosophila Stock Center (BDSC): *w*^*1118*^, UAS-GFP.nls (#4775) and dTet-Gal4 [24]. Flies crosses were performed on standard cornmeal-agar medium at 25°C.

### Transgenic fly generation

cDNA of mouse methyl cytosine dioxygenase TET2 in a mammalian expression vector pcDNA3-mTET2 (5763 bp) was obtained from (Addgene: Plasmid number 60,939). TET2-wt, TET2-R43G, and TET2-R1261C were then placed into fly cloning and transformation vector pUASTattB [25] containing an N-terminal 1×Flag-tag (DYKDDDDK). Cloning and site-directed mutagenesis was performed by Custom DNA Constructs LLC. All transgenes of mTET2 wild type and mutants are generated using PhiC31 (φC31) integrase system using the fly line BDSC (#8621) with a landing site on chromosome 2 (Chr 2, 55C4, 2 R:18357321).

UAS-dTet flies were generated by synthesizing the cDNA of *Drosophila* Tet (8829 bp) (NM_001274415.1) and cloning it into *Drosophila* specific cloning vector pUASTattB containing N-terminal 1×Flag-1×HA tags (LDGGYPYDVPDYAGGLD) (MDYKDDDDK) within (XbaI and KpnI) sites. dTet synthesis and cloning were performed by GenScript, USA, Inc. Transgenic dTet flies are generated using PhiC31 (φC31) integrase system using the fly line BDSC (#8621) with a landing site on chromosome 2 (Chr 2, 55C4, 2 R:18357321). All transgenes were generated by BestGene Inc.

### RNA extraction and quantitative RT-PCR

Total RNA was extracted from 1-day old adult fly heads using the TRI Reagent (Sigma-Aldrich). Approximately 100 µL of Trizol were added to samples followed by manual homogenization with a pestle (brains) or with a Tissue Lyser (whole larvae, Jingxin). Samples were incubated at room temperature for 5 min followed by centrifugation at 21,000 g for 5 min at 4°C. Chloroform (Sigma-Aldrich) was subsequently added and samples were vortexed and then centrifuged at 21,000 g for 10 min at 4°C. The supernatant containing RNA was collected in a new tube and cold isopropanol was added. Samples were kept at room temperature for 10 min to allow for maximum RNA precipitation prior to centrifugation at 21,000 g for 20 min at 4°C. The RNA pellet was subsequently washed with 70% ethanol twice and centrifuged at 21,000 g for 10 min each at 4°C. The ethanol was discarded and the pellet was air-dried on ice before being resuspended in nuclease free water. Genomic DNA was removed from extracted RNA by incubating samples with DNase (ThermoScientific) for 1 h at 37°C. cDNA synthesis was performed using the Revert Aid First Strand cDNA Synthesis Kit (ThermoScientific). Quantitative RT-PCR reactions were performed in triplicates on Biorad CFX Connect using SYBR Green (BioRad SSO Advanced Universal SYBR Green Supermix). Samples were normalized to *rp49* and relative gene expression was calculated using the Livak method. Statistical significance was measured using an unpaired student’s *t-*test. P-values less than 0.05 were considered statistically significant. Primer sequences can be found in **Supplemental Table S1**.

### Western blot

Twenty adult heads were collected in 2× Laemmli buffer containing 4% protease inhibitor (Roche) and 10% phosphatase inhibitor (Roche). Samples were homogenized using a pestle followed by sonication for 10 min at 4°C. Samples were then centrifuged for 15 min and the supernatant was collected. 100 µg of protein were loaded onto 8% acrylamide gel and were run at 90 V. Precision Plus Protein Kaleidoscope ladder (Biorad) was used as a molecular weight marker. The gel was removed and placed on a PVDF membrane (Biorad) inside a transfer apparatus for blotting overnight at 30 V at 4°C. The membrane was then placed in a blocking solution (5% milk in PBS-Tween 0.05%) for 1 h. The primary antibody was added overnight in blocking solution (anti-β actin, Abcam, 1:5000; anti-Flag, Sigma, 1:500). The membrane was washed three times in PBS-Tween for 10 min each and was then incubated with an HRP-conjugated secondary antibody (Abcam, 1:5000) for 2 h at room temperature. The membrane was washed three times with PBS-Tween for 10 min each. Imaging was done on the Chemidoc MP machine using ECL Clarity Max (Biorad).

### Transcriptome sequencing (RNA-seq)

Total RNA was collected from thirty 1-day old male and female fly heads (in equal ratios) by Trizol (Sigma-Aldrich) extraction as previously described. Twelve RNA samples in ethanol (three biological replicates for each group) were sent to Macrogen (Seoul, South Korea) for further processing. Ribodepletion was performed on the samples (Illumina TruSeq Stranded Total RNA with Ribo-Zero Gold Human/Mouse/Rat) followed by sequencing using Illumina TruSeq on the NovaSeq6000 platform with a scope of 4GB of reads per sample. Fastq files were provided with each containing around 25 million paired end reads of a mean read length of 100 basepairs (bp).

### Bioinformatics analysis

Data was prepared by performing quality control (QC) checks on all fastq files to ensure reads were of good quality and adapter-free using fastqc (v0.11.8) and results were aggregated using multiQC (version 1.7). Alignment to reference genome dmel_r6.28_FB2019_03 obtained from flybase (https://flybase.org/) was performed using hisat2 (version 2.1.0) with the following parameters: –new-summary -p 50 –RNA-strandness RF –dta –max-intronlen 300,000. The generated SAM files were transformed to BAM, sorted, and indexed using samtools (version 1.4.1). Normalized bigwig files used for visualizations on the Integrative Genomics Viewer (version 2.4.16) were generated using deeptools (version 3.1.0–2-38cfe39) with the following parameters: -p50/–normalizing CPM. Additional quality checks were performed on the generated bam files (gene body coverage, correlation matrix between biological replicates, PCA analysis between conditions, etc.) using deeptools and RSeQC package (version 2.6.4). Gene expression counts matrix was generated using featureCounts (version 1.6.0) with the following parameters: -s 2 -p -t gene -g gene_id -T 50. Starting from the generated counts matrix, differential expression analysis was done in R (version 3.6.1) using DESeq2 package (version 1.24.0). Genes with an expression sum across all replicates and conditions that were less than 10 reads were removed from the counts matrix as per recommendations from DESeq2 authors. DESeq2 was used with default parameters. Genes with abs (log2 fold-change) > = 1 and p-adj < = 0.05 were considered as differentially expressed between conditions. Gene ontology analysis for the differentially expressed genes was done on the FlyMine platform (v48, 2019).

### Drosophila activity assay

To record the daily activity and sleep of wild type and mTET2 mutants, the *Drosophila* activity monitor (DAM) system was used (TriKinetics Inc). Glass activity tubes containing 5% sucrose and 2% agarose, sealed with melted paraffin at the food end, and plugged with cotton at the other end were used. Male flies were loaded into the activity tubes with one fly per tube. The tubes were placed in monitors with each monitor corresponding to a genotype. The following stocks were obtained from Vienna Drosophila RNAi Center: Atg16-RNAi (#25651), ich- RNAi (#20127), and UAS-GFP-RNAi (Kind gift from Dr. Martin Hasselblatt).

The following genotypes were used for this analysis (**Supplemental Table S2**): w^1118^ (*n* = 24), dTet-Gal4>wt-mTET2 (*n* = 24), dTet-Gal4>mTET2-R43G (*n* = 28), dTet-Gal4>mTET2-R1261C (*n* = 32), dTet-Gal4>wt-mTET2-Atg16 RNAi (*n* = 21), dTet-Gal4>mTET2-R43G-Atg16 RNAi (*n* = 24), dTet-Gal4>wt-mTET2 ich RNAi (*n* = 20), and dTet-Gal4>mTET2-R43G-ich RNAi (*n* = 20), dTet-Gal4>dTet (*n* = 21), dTet-Gal4>wt-mTET2-dTet RNAi (*n* = 29), dTet-Gal4>Atg16 RNAi (*n* = 19), dTet-Gal4>ich RNAi (*n* = 25), dTet-Gal4 (*n* = 14). The activity monitors were connected to a data collection system and the experiment was run for 40 days. The DAM system was maintained at 25°C, at a humidity above 60%, at a cycle of 12 hours of daylight and 12 hours of dark. Each tube was placed into a channel in which an infrared light beam detects the movement of the fly. Trikinetics data acquisition software (DAMSystem308, Trikinetics Inc.) saves the data as activity of each fly per 5 minutes. Locomotor activity, sleep duration, and survival of the flies were analysed by considering 5 minutes of inactivity as sleep and more than 24 hours as death. Data analysis was performed on the living flies over 30 days [26]. ActogramJ plug-in (v0.9) was used to draw periodic actograms for each fly [27]. Calculation and statistical analysis of data were carried out using Microsoft Excel and Graphpad Prism (Graphpad Software, La Jolla, CA, USA).

### Immunofluorescence staining

Adult brains were dissected in PBS with 0.3% Triton X-100 (PBST, Sigma-Aldrich) and were then fixed in 4% formaldehyde for 20 min at room temperature. Brains were then washed in PBST three times for 20 min each. Subsequently, brains were placed in blocking solution (5% normal goat serum [Dako] in PBST) overnight at 4°C. Samples were then incubated in primary antibody diluted in blocking solution overnight at 4°C (mouse anti- Repo from Developmental Studies Hybridoma Bank (DSHB) 8D12, 1:30). Brains were then washed in PBST 3 times for 20 min each and were then incubated with fluorochrome-conjugated secondary antibody (AlexaFluor-594 anti-mouse- Abcam, 1:500) for 2 h. Samples were then incubated in DAPI solution (1:5000, 10^−3^mg/mL, Molecular Probes) for 5 min and were then washed in PBST three times for 20 min each. Samples were then mounted onto microscope slides with gold anti-fade solution (Invitrogen) for subsequent analysis using the Zeiss LSM 710 laser scanning confocal microscope. All images were acquired and analysed using the Zeiss ZEN 9 image software.

### Dot blot

RNA samples were dotted onto a nylon membrane (Sigma-Aldrich). The membrane was then stained with methylene blue (Alfa Aesar) for visualization of loading (1% in 0.3 M sodium acetate). The membrane was washed with distilled water and was subsequently blocked in 5% milk in PBS-Tween (0.05%) for 1 h at room temperature. The membrane was then incubated with rabbit anti-6 mA for DNA (Synaptic Systems, 1:1000) and rabbit anti-5hmC for RNA (Abcam, 1:5000) overnight at 4°C. The following day the membrane was washed 3 times with PBS-Tween and was subsequently incubated with a rabbit HRP-conjugated secondary antibody (Santa Cruz, 1:5000) for 2 h at room temperature. The membrane was washed 3 times and was then imaged using the Chemidoc MP machine.

### Survival analysis

Twenty third-instar larvae were collected for each genotype and were placed into new vials and the number of pupae and adults was recorded. The survival assay was performed three times (total of 60 larvae) and a two-tailed student’s t-test was used to determine significance between groups.

### Gap measurement

To detect thorax and abdomen gaps, adult flies were collected and placed at −20ºC for 1 h. Subsequently, wings and legs were removed to allow for proper positioning and imaging. Flies were placed ventrally on double-sided tape and images were taken of the thorax and abdomen of each fly using the Olympus SZX10 upright microscope. The space between the two bilateral sides of the thorax and abdomen was measured using the Olympus CellSens software.

### Haemocyte count in third instar larvae

To count the number of haemocytes, third instar larvae of the following genotypes were used (**Supplemental Table S2**): UAS-Relish-RNAi (Vienna Stock Center VDRC 49414), UAS-Myd88 RNAi (Vienna Stock Center VDRC 25399), UAS-GFP-RNAi (Kind gift from Martin Hasselblatt), UAS-Stat92E RNAi (Vienna Stock center VDRC106980), dTet-Gal4>w^1118^ (*n* = 30), dTet-Gal4>wt-mTET2 (*n* = 30), dTet-Gal4>mTET2-R43G (*n* = 30), dTet-Gal4>mTET2-R1261C (*n* = 34), dTet-Gal4>wt-mTET2-Stat92E RNAi (*n* = 31), dTet-Gal4>mTET2-R1261C-Stat92E RNAi (*n* = 30), dTet-Gal4>wt-mTET2-Myd88 RNAi (*n* = 29), dTet-Gal4>mTET2-R1261C-Myd88 RNAi (*n* = 32), dTet-Gal4>wt-mTET2-Relish RNAi (*n* = 34), dTet-Gal4>mTET2-R1261C-Relish RNAi (*n* = 36), dTet-Gal4>wt-mTET2-GFP RNAi (*n* = 30), and dTet-Gal4>mTET2-R1261C-GFP RNAi (*n* = 29). Third instar larvae were collected from each cross and washed with PBS 1 × . The haemolymph of each larva was bled in 10 μL of PBS 1× then added to a volume of 10 μL of trypan blue. The total bleed volume was transferred to a haemocytometer and cells were counted. Graphs and statistical evaluation were performed using GraphPad Prism version 8.4.2.

## Results

### Adult flies expressing wild type or mutant mTET2 show survival and morphological defects

**Figure 1.**
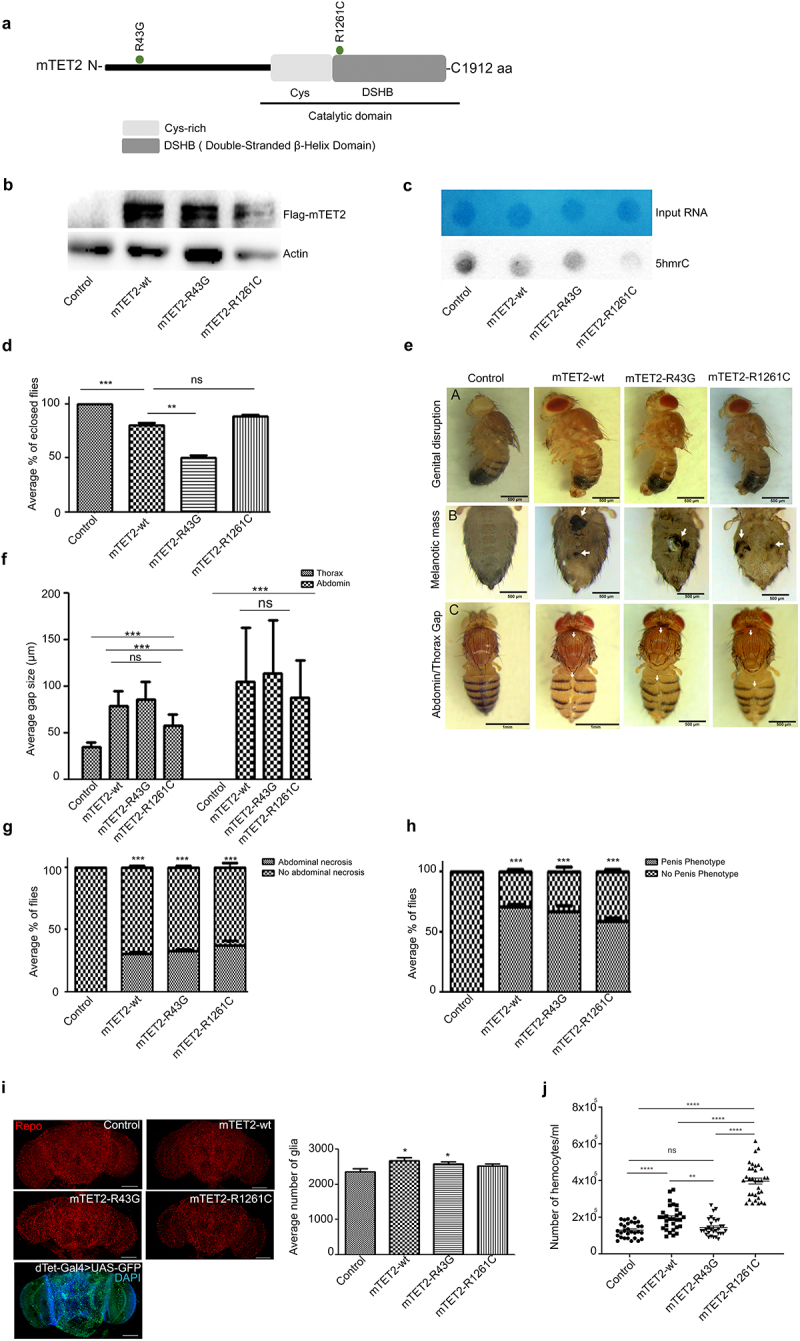
**Morphological and survival defects exhibited by mTET2 expressing flies**. (**a)** Schematic representation of mTET2 with its functional domains. Mutations R43G at the N-terminus and R121C at the catalytic domain are depicted. Transgenes tested in the study in dTet-expressing cells using the dTet promoter (dTet-Gal4) are indicated in (**Supplemental Figure S1**). (**b**) Validation of transgene expression in flies by Western blot against Flag tag (*n* = 3, 20 adult heads per group). (**c**) the expression of mTET2 is associated with an alteration in levels of 5hmc on RNA (5hmrC). Representative images from dot blot assays on 5hmrC abundance in adult heads with quantification of signal intensity normalized to loading control and shown as relative to control. Methylene blue (MB) was used as a loading control. 900 ng of RNA were loaded. (**d**) the average percentage of third instar larvae that eclosed as adults (*n* = 3, 30 larvae per group). Mean is shown with s.e.m. (**e-panel A**) Light microscopy images of male flies showing the malformation and protrusion of male genitalia in mutant flies as compared to the wild type control. Scale bar, 500 μm. (**e-panel B**) Light microscopy images of flies showing the presence of melanotic masses in the abdomen of transgenic flies that are not found in the wild type controls. Scale bar, 500 μm (second panel). (**e-panel C**) Light microscopy images of adult flies showing gaps along the midline in the thorax and abdomen (arrows). (**f**) Quantification of the average size of thorax and abdomen gaps in adult flies (*n* = 3, 20 flies per group), (**g**) Quantification of the occurrence of the melanotic masses in adult flies (*n* = 3, 20 flies per group), (**h**) Quantification of the occurrence of penis malformation in males (*n* = 3, 10 flies per group) (**i**) Representative confocal maximum intensity projections of adult brains stained with the glial marker Repo. Scale bar, 100 μm. Quantification of the average amount of glia per brain as calculated by ImageJ’s ITCN tool (*n* = 30 per genotype). Mean is shown with s.e.m. Confocal maximum intensity projection of Tet-Gal4>UAS-nuclear GFP brains showing the pattern of cells in which expression of transgenes is driven. Scale bar, 100 μm (lower panel). Control group used is a driver control dTet-Gal4>w^1118^); (**j)** Number of haemocytes present in larval haemolymph expressing either no transgene (driver control; dTet-Gal4>w^1118^) (*n* = 30), mTET2-wt (*n* = 30), mTET2-R43G (*n* = 30), or mTET2-R1261C (*n* = 34) with mTET2-wt and mTET2-R1261C showing a remarkable increase (n ≅ 30 in each genotype). *p < 0.05, **p < 0.01, ***p < 0.001 and ****p < 0.0001.

To better understand the role of mTET2 in development, we opted to express two mTET2 mutations in dTet expressing cells in *Drosophila*: the Arginine 43 to Glycine (R43G) mutation within the N-terminus of TET2 (previously found in a DIPG sample as part of a whole-exome sequencing study) and the Arginine 1261 to Cysteine (R1261C) mutation within the catalytic domain that is commonly found in AML patients [27] (Figure 1a, Supplemental Figure S1).

The presence of transgenes was confirmed and their catalytic activity was assessed (Figure 1b,c). As predicted, only the mutation that is present within the catalytic domain of mTET2 (R1261C) showed reduced levels of 5hmC in RNA (5hmrC) relative to control and mTET2-wt flies (Figure 1c). Next, we carried out a survival analysis following larval to adult stages in mTET2 expressing flies. The expression of both wild type and mutant mTET2 (R43G or R1261C) in dTet-expressing cells resulted in reduced survival of adult flies (Figure 1d). The expression of mTET2-R43G showed the highest proportion of lethality with only around 50% of larvae reaching the adult stage (*p* = 0.0018), as compared to 88% survival in flies expressing the mTET2-R1261C, which was non-significant compared to mTET2-wt. Expressing mTET2-wt on the other hand caused a milder lethality with reduced survival (80%, *p* = 0.0023). Furthermore; to assess whether overexpression of *Drosophila* dTet will lead to similar effect in survival, we have expressed dTet in dTet-expressing cells. Few pupae eclosed to adult flies (*p* < 0.0001) in comparison to mTET2 indicating more severe effect on survival upon dTet expression **(Supplemental Figure S2**).

Eclosing flies exhibited severe defects in thoracic and abdominal morphology. Gaps were seen along the midline between the two sides of the body, which is indicative of a defect in the process of closure that must occur during development and specifically during morphogenesis in the pupal stage (Figure 1e, panel C). This effect was seen in 100% of flies expressing wild type or mutant mTET2 but showed varying levels of severity (Figure 1f). It is important to note that the variations in severity levels between all mTET2 expressing flies could be dependent on the dose of transgene expression that might vary between different mTET2 expressing flies. Of note, some of the control flies (Tet-Gal4>w^1118^) also showed some gaps in the thorax. Measurements of the thorax and abdomen gaps showed that there was no major difference between the mTET2-wt and mutant groups (Figure 1f). In addition to the thorax and abdomen phenotype, some adult flies exhibited melanotic masses on the ventral aspect of the abdomen (Figure 1e, panel B). This was found in around 31–33% of flies expressing mTET2-wt or mTET2-R43G, and in 20% of flies expressing mTET2-R1261C (Figure 1g). Moreover, the genitalia in transgenic male flies were structurally altered whereby they protruded outside the body and formed an irregular floral-like structure (Figure 1e, panel A). Quantification of this phenotype showed that it was highly prevalent and present in around 60–70% of male flies (Figure 1h). We further assessed the survival in flies overexpressing mTET2-wt in *Drosophila* Tet (dTet) knock down background (dTet RNAi) to examine whether the effect of mTET2 expression will be modulated upon endogenous dTet knockdown. The survival data showed a slight reduction in the number of enclosed pupae in mTET2-wt overexpression with dTet RNAi knockdown compared to mTET2-wt expression alone (*p* = 0.0122) **(Supplemental Figure S2)**. Taken together, these findings suggest that ectopic expression of wild type or mutant mTET2 induces defects in survival and morphological body structure with varying severity.

### Expression of wild type or mutant mTET2 leads to an increase in the number of glia and haemocytes in fly brain and larvae

Since our interest was to determine whether the N-terminal mTET2-R43G mutation has any impact on brain development and function, it was crucial to determine whether the expression of this mutation affected adult fly brains. While no obvious gross morphological changes were observed in the fly brains, there was a significant increase in the number of Repo-positive differentiated glial cells (~2583.59 glial cells) compared to the control (~2361 glial cells, *p* = 0.045) (Figure 1i). The increase in cells was most apparent in the central brain region. As seen in the previous phenotypes, the expression of mTET2-wt alone also induced a similar expansion in the differentiated glial population (~2666.3 glial cells) with respect to the control (~2361 glial cells, *p* = 0.0254). To confirm whether the transgene is expressed in the same cell populations in which the increase in glia is seen, GFP-driven expression of dTet was analysed and the pattern of expression appeared to be in the central brain region and optic lobes, consistent with the brain areas where the glial increase was observed (Figure 1i). Of note, brains from flies expressing mTET2-R1261C, which is the mutation found in leukaemia, did not show any increase in the number of glia relative to control brains (Figure 1i). We therefore chose to evaluate the status of the haematopoietic system. Indeed, larvae expressing mTET2-R1261C showed a significant increase in haemocyte numbers (3.97×10^5^/mL) as compared to mTET2-wt (1.94×10^5^/mL, *p* < 0.0001) and control larval haemolymph (1.29×10^5^/mL, *p* < 0.0001) (Figure 1j). Nevertheless, mTET2-R43G expression did not lead to a remarkable difference in larval haemocyte numbers (1.43×10^5^/mL) compared to the control (1.29×10^5^/mL) (Figure 1j). Importantly, mTET2-wt larvae also displayed a similar phenotype with a significant increase in the number of haemocytes (1.94×10^5^/mL) with respect to the control (1.29×10^5^/mL, *p* < 0.0001). The obtained findings indicate that the expression of mTET2-R1261C leukaemia mutation in flies triggers the highest increase in haemocyte numbers in the context of the haematopoietic system and that ectopic expression of TET2 – either wild type or mutant – causes defects both at the level of haemocyte and glial cell counts.

### Expression of mTET2-R43G and R1261C mutants leads to shorter lifespan and defects in fly activity and sleep patterns

**Figure 2.**
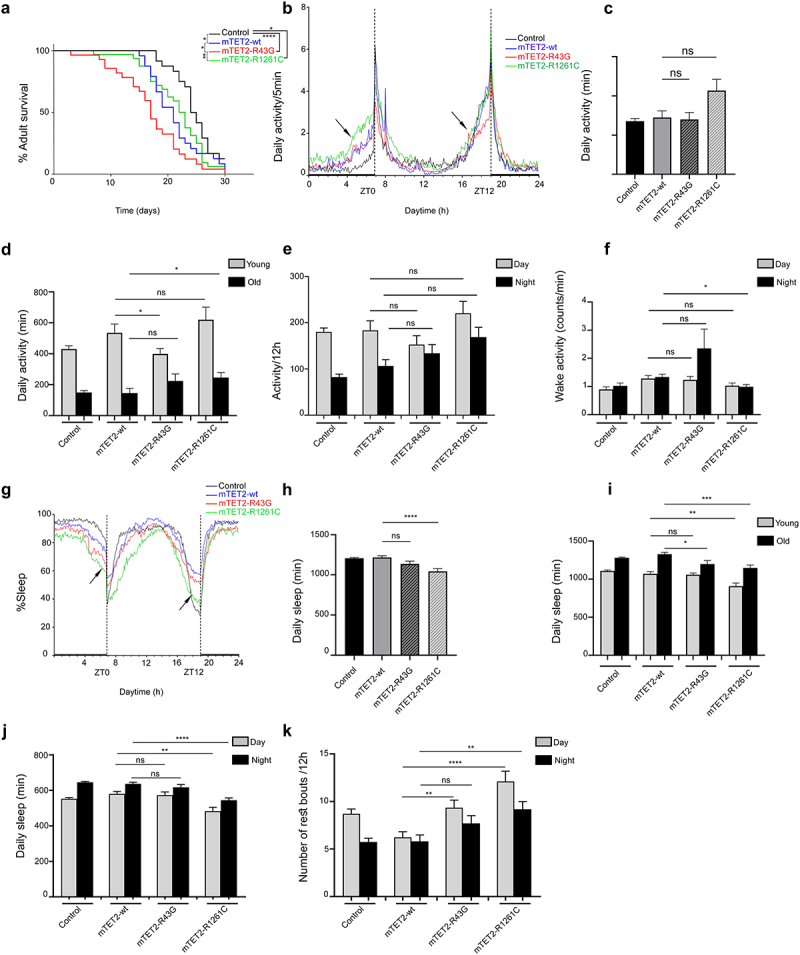
**Expression of human mTET2-R43G and mTET2-R1261C in dTet expressing flies results in altered lifespan and circadian phenotypes**. (**a**) Kaplan-Meier survival curve of male flies expressing either no transgene (*w*^*1118*^ control, *n* = 24), mTET2-wt (*n* = 24), mTET2-R43G (*n* = 28), or mTET2-R1261C (*n* = 32) driven by dTet-Gal4. Statistical significance of the difference between survival curves was determined using the Mantel-Haenszel test. Survival of mTET2-wt, mTET2-R43G, and mTET2-R1261C was significantly reduced compared to control (* *p* < 0.05, ****p < 0.0001). Survival of mTET2-R43G flies was significantly reduced compared to mTET2-wt and mTET2-R1261C (Unpaired t-test; *p < 0.05, **p < 0.01). (**b**) Locomotor activity graphs were analysed over 30 days. For each group, the locomotor activity levels of individual male flies were measured in 5-minute bins and then averaged to obtain a representative activity profile. *Drosophila melanogaster* generally exhibits two activity bouts: one centred around ZT0 (morning peak) and the second around ZT12 (evening peak), arrows indicate the anticipatory activity prior to light transition states. (**c**) Graph showing the average activity of the flies over 24 h intervals (Unpaired t-test; ns: *p* > 0.05). (**d**) the flies are subdivided into two groups in an age-dependent manner as locomotion is age-dependent: ‘young’ represents 1–13 days old and ‘old’ represents 14–30 days old. Note that mTET2-R43G young flies are significantly less active (* *p* < 0.05), whereas old mTET2-R1261C flies are more active (* *p* < 0.05) compared to mTET2-wt. (**e**) Graph showing the average locomotor activity over 12 h intervals (day: light on, night: light off) (Unpaired t-test; ns: *p* > 0.05). (**f**) Graph illustrating the wake activity counts per min over 12 h intervals. The wake activity is a measure of the activity rate when the flies are awake. Note that there is a significant decrease in the wake activity in mTET2-R1261C flies compared to mTET2-wt flies (*p < 0.05). (**g**) Graph illustrating the percentage of time that flies spent sleeping over several days. For each group, the percentage of flies sleeping was measured in 5-minute bins and then averaged to obtain a representative sleep profile. (**h**) Graph showing the average sleep of the flies over 24 h intervals. ZT0 indicates the morning peak and ZT12 the evening peak. Note that there is a significant decrease in the average of sleeping in mTET2-R1261C flies compared to mTET2-wt flies (****p < 0.0001). (**i**) Young and old mTET2-R1261C flies and old mTET2-R43G flies sleep significantly less compared to mTET2-wt flies (Unpaired t-test; * *p* < 0.05, **p < 0.01, ***p < 0.001). (**j**) Graph showing the average of daily sleep minutes for all flies over 12 h intervals over 30 days. During day and night, mTET2-R1261C expressing flies showed a significant decrease in sleep time compared to mTET2-wt flies (Unpaired t-test, ** *p* < 0.01, *** *p* < 0.001). (**k**) Graph showing the average number of rest bouts for all flies in one group for 12 h intervals over 30 days. During the day, mTET2-R43G flies showed significantly more rest bouts compared to mTET2-wt flies (Unpaired t-test, ** *p* < 0.01). During the day and night, mTET2-R1261C expressing flies showed significantly more rest bouts compared to mTET2-wt flies (Unpaired t-test ***p < 0.001, ** *p* < 0.005). (For all figures, ns: *p* > 0.05).

Given the increase in the number of differentiated glial cells in brains of mTET2 expressing flies and the known role of dTet in glial cell organization and function in axonal targeting [28], we performed behavioural assays using the *Drosophila* activity monitor system to investigate the impact of R43G or R1261C expression driven by dTet-Gal4 on lifespan as well as circadian and locomotor behaviour in adult flies. Consistent with the survival analysis performed to quantify the number of flies eclosing to adults, mTET2-R43G mutants showed an overall decrease in adult lifespan where flies tend to die in the first 20 days (Median survival = 17 days) compared to mTET2-wt (Median survival = 21, *p* = 0.0371) and mTET2-R1261C mutants (Median survival = 22.5 days, *p* = 0.0049) (Figure 2a). mTET2-R1261C mutants, on the other hand, showed a minimal decrease in survival (Median survival = 22.5 days) compared to the control (Median survival = 24.5 days, *p* = 0.0446) that was not significant when compared to mTET2-wt mutants (median survival 21, *p* = 0.544) (Figure 2a). mTET2-wt flies also showed an overall reduced lifespan (Median survival = 21) when compared to the wild type control (Median survival = 24.5). To make sure the phenotype observed in mTET2-wt is not from the driver itself, we performed survival analysis on dTet-Gal4 flies. As expected, lifespan of dTet-Gal4 driver (control) (Median survival = 23) was comparable to the wild type control (Median survival = 24.5) indicating no effect for the driver alone on fly survival **(Supplemental figure S3a**). Next, we assessed whether overexpression of *Drosophila* Tet (dTet) may result in similar effects on survival. Notably, dTet overexpression was very severe where median survival of these flies was 15 days **(Supplemental figure S3a**). In addition, severe life span reduction was observed in adult mTET2-wt flies in dTet knockdown background compared to mTET2-wt expression alone indicating that loss of endogenous dTet exacerbated mTET2 overexpression survival phenotype **(Supplemental Figure S3b)**.

Generally, *Drosophila* exhibits a bimodal (double-peaked) activity pattern under light/dark conditions with morning and evening activity bouts that are separated by a ‘siesta’ [29]. Circadian analysis in mTET2 mutants and control flies revealed a daily locomotor activity with two major activity bouts peaking at ZT0 (morning peak) and the second ZT12 (evening peak) (Figure 2b). In addition, activity during the anticipation phase before the dark-to-light transition (ZT0) tended to be highest in mTET2-R1261C mutants whereas this increase in locomotor activity was similar in mTET2-R43G and mTET2-wt flies, which was higher than the control (Figure 2b). Before the light-to-dark transition (ZT12), mTET2-R43G mutants showed a decline in the activity during the anticipation phase compared to mTET2-wt and control flies (Figure 2b). These findings indicate that expression of mTET2 mutations may interfere with the proper locomotor activity during morning and evening anticipation and proper circadian functioning. While the mean daily activity over 12 h or 24 h intervals was not significantly different between the groups (Figure 2c,e), analysis of daily locomotor activity conducted in young (1–13 days) versus old flies (14–30 days) revealed remarkably diminished levels of activity in young mTET2-R43G flies, as confirmed by the actogram (Figure 2d, Supplemental Figure S4). This effect was most apparent prior to ZT12 (Figure 2b). This activity, however, was higher and more evident in old mTET2-R1261C mutants (Figure 2d, Supplemental Figure S4) compared to mTET2-wt flies prior to ZT0 (Figure 2b). This suggests that the expression of either mTET2 mutation interferes with locomotor behaviour in an age-dependent manner in flies. Furthermore, the level of activity when flies are awake, known as the wake activity, was significantly less during the night in mTET2-R1261C mutants compared to mTET2-wt flies (Figure 2f), whereas mTET2-R43G mutants did not show any significant variation. Alongside the activity and locomotor parameters, changes in sleep behaviour were monitored and the average amount of daily sleep was significantly reduced in mTET2-R1261C but not in mTET2-R43G mutants compared to mTET2-wt flies (Figure 2g,h). This effect was more prominent in young and old mTET2-R1261C flies (Figure 2i) during both light and dark phases (Figure 2j). Conversely, only old mTET2-R43G mutants displayed less amount of total sleep compared to mTET2-wt flies (Figure 2i). Accordingly, the number of rest bouts, defined as five minutes with no activity, was significantly higher in mTET2-R1261C mutants during both day and night (Figure 2k). This effect was prominent during the day in mTET2-R43G mutants compared to mTET2-wt flies (Figure 2k). This indicates that mTET2-R43G flies may exhibit shorter light sleep phases, whereas mTET2-R1261C flies display shorter light and dark sleep phases. Altogether, our data suggest that ectopic expression of mTET2-R43G or R1261C impaired lifespan, locomotor activity, and sleep/rest behaviour. Although the overall daily activity of mTET2 mutant flies did not vary, the locomotor behaviour was impaired in an age-dependent manner.

### Expression of wild type or mutant mTET2 results in changes in gene expression

**Figure 3.**
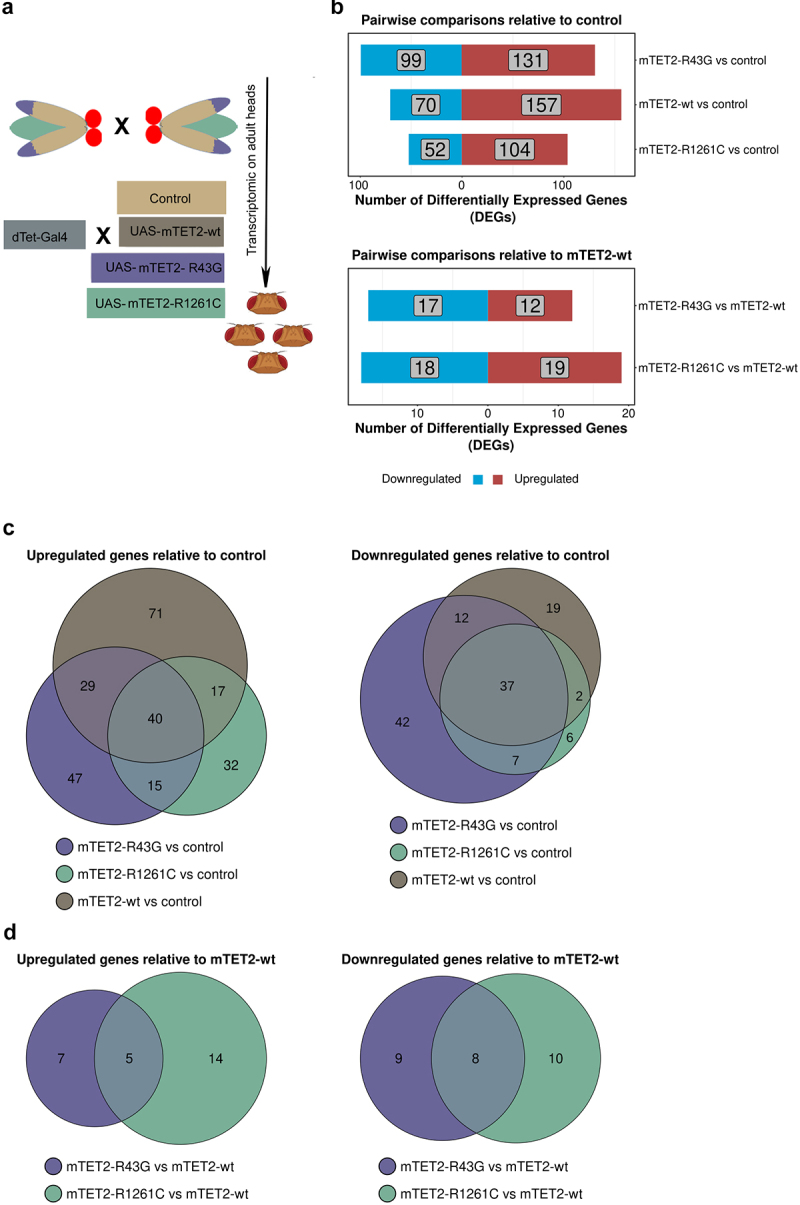
**Differentially expressed genes in flies expressing mammalian TET2 transgenes relative to control and mTET2-wt**. (**a**) Schematic of flies used in the transcriptomic analysis. (**b**) Several differentially expressed genes (DEGs) were identified in each group relative to dTet-Gal4>w^1118^ (control) and mTET2-wt (lower panel). Blue genes are downregulated, red genes are upregulated. (**c-d**) Venn diagram of the DEGs that are common and different between the groups when compared to dTet-Gal4>w^1118^ (control) and mTET2-wt. (**c**) Depicts up- and down-regulated genes compared to dTet-Gal4>w^1118^ (control) (**d**) Depicts up- and down-regulated genes compared to mTET2-wt.

As TET proteins are known to play a role in gene expression due to their activity as epigenetic regulators, we sought to identify whether expression of wild type or mutant mTET2 in the fly brain was associated with gene expression changes and to better understand whether this was specific to the mutants. To this end, we performed RNA-sequencing (RNAseq) on adult heads from controls and flies expressing mTET2-wt, mTET2-R43G, or mTET2-R1261C (Figure 3a). The number of differentially expressed genes (DEGs) in all pairwise comparisons relative to control or to mTET2-wt is summarized in Figure 3b. Upon expression of mTET2-wt, mTET2-R43G, or mTET2-R1261C, more genes were significantly upregulated than those that were downregulated relative to controls (Figure 3b). The total number of dysregulated genes was similar between mTET2-R43G and mTET2-wt (230 vs. 227 DEGs, respectively). Interestingly, a smaller number of genes was affected by the catalytic inactive mutant mTET2-R1261C (156 DEGs) (Figure 3b). When comparing all three groups to controls, we found a relatively high number of genes that were commonly upregulated and downregulated (40 and 37 DEGs respectively) (Figure 3c). This may indicate that the wild type mammalian TET2 might exert some gene expression changes and that the dosage of TET2 is crucial for gene expression. We further compared DEGs between the two mutants mTET2-R1261C and mTET2-R43G to mTET2-wt to determine the gene expression changes that were unique to the mutants (Figure 3d). The number of DEGs was much smaller in this comparison: 29 genes were dysregulated in mTET2-R43G flies (12 upregulated and 17 downregulated) (Figure 3d), and 37 genes were dysregulated in mTET2-R1261C flies (19 upregulated and 18 downregulated) (Figure 3d). Thirteen DEGs were commonly found in both mTET2-R1261C and mTET2-R43G, of which 5 were upregulated and 8 were downregulated (Figure 3d).

### Expression of wild type or mutant mTET2 leads to upregulation of genes involved in innate immune pathways in *Drosophila*

**Figure 4.**
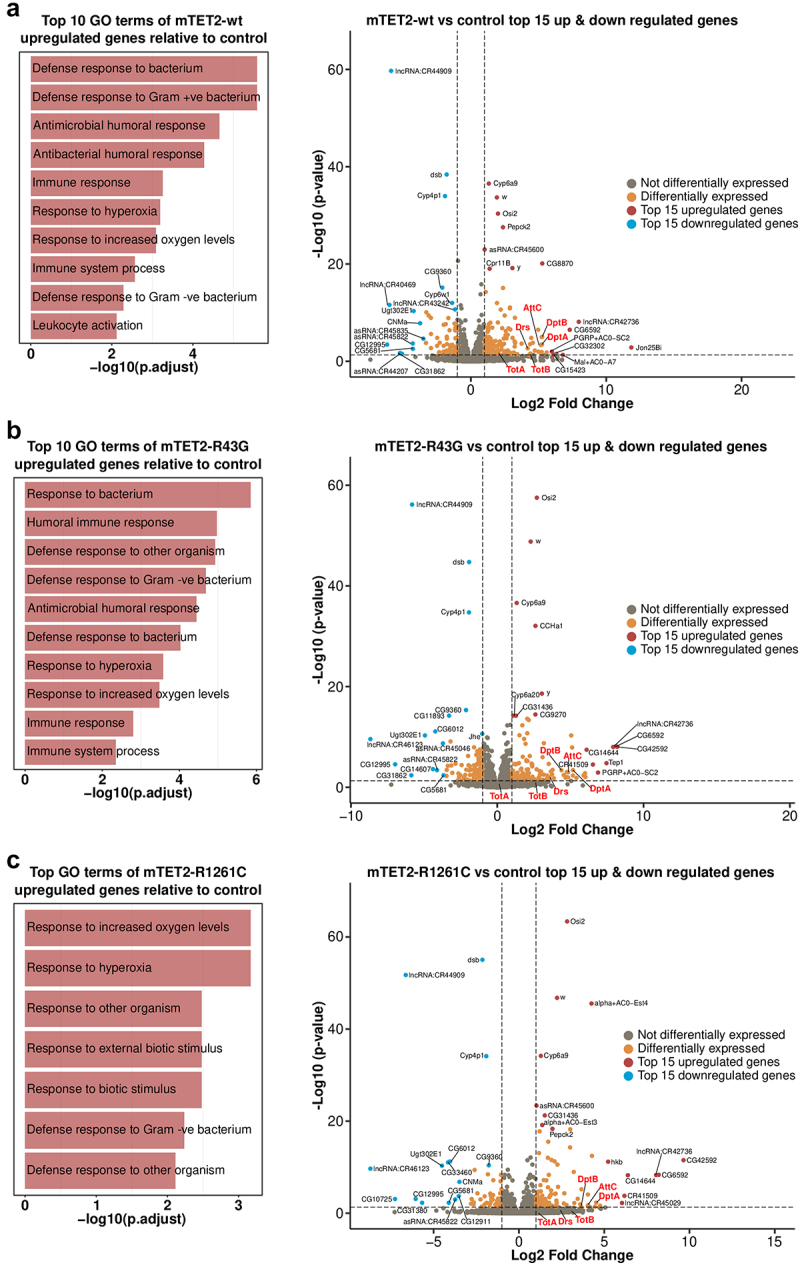
**Gene ontology of genes that are upregulated in all mTET2 expressing flies versus controls**. Gene ontology of top 10 upregulated DEGs that were identified in mTET2 heads versus controls, with most being involved in immune related pathways. Volcano plot indicating top 15 genes upregulated and downregulated relative to control group.

To gain a better understanding of cellular and molecular pathways that may be affected by these changes in gene expression, we performed a gene ontology enrichment analysis to identify pathways that were significantly dysregulated in all mTET2 transgenic flies. To our surprise, the only pathways that were commonly deregulated across all groups included those involved in immune defence to gram negative and gram-positive bacteria, response to hyperoxia, and response to high oxygen levels, with these pathways pertaining to genes that were significantly upregulated (Figure 4a–c). This was consistent with our further detailed analysis of top-15 genes that were significantly upregulated and downregulated in all groups expressing mTET2 versus the control with a LogFC > = 1 and p-adjusted value < = 0.05 considered as differentially expressed. In addition, several long non-coding RNAs were upregulated in all mTET2 expressing groups including LncRNA: CR42736 (LogFC = 7.97 in mTET2-wt, 7.92 in mTET-R43G, and 8.04 in mTET2-R1261C). Several non-annotated genes were also among the top upregulated genes. These include CG42592 (upregulated in mTET2-R43G and mTET2-R1261C) with unknown function and CG6592 (upregulated in all three transgenic groups) which is another serine-type endopeptidase involved in proteolysis. Most of the top-15 downregulated genes were non-annotated genes with few long non-coding RNAs (Figure 4a–c, Supplemental Table S3). Notably, when assessing the DEGs that were differentially regulated between flies expressing mTET2-wt, mTET2-R43G, or mTET2-R1261C mutants versus controls, we noticed that many genes involved in the innate humoral immune pathways were upregulated (Figure 4a–c, Volcano plots, Supplemental Table 3). These included antimicrobial peptides such as *Drosomycin* (*Drs*), *Diptericin* (*Dpt*), *Attacin* (*Att*), as well as *Turandot A* and *B* which are products of the JAK-STAT pathway. There are three main humoral immune pathways in flies: the Toll pathway, the immune deficiency (IMD) pathway, and the JAK-STAT pathway. These pathways mainly differ in the type of microorganisms that activate them (bacterial, fungal, or viral), although some overlap has been shown to exist. The genes that are transcribed in response to these pathways, *Drosomycin* (*Drs*), *Diptericin* (*Dpt*), *Attacin* (*Att*), and *Turandot* (*Tot*), were significantly upregulated in the different pairwise comparisons relative to control (Figure 4a–c, Supplemental Table S3). For example, DptA was significantly upregulated with LogFC = 5.25, 5.17, and 4.52 in mTET2-wt, mTET2-R43G and mTET2-R161C respectively. A similar but milder increase in *Drs* expression was seen in mTET2-wt, mTET2-R43G, and mTET2-R161C (LogFC = 4.19, 3.56, and 2.41, respectively). Quantitative real-time PCR (qRT-PCR) validation experiments were almost completely in line with the data obtained from the RNA-seq analysis (**Supplemental Figure S5a-d**). *Drosomycin* showed a significant increase in mTET2-wt and mTET2-R1261C flies relative to controls, but not in mTET2-R43G, although there was a tendency towards increased expression (**Supplemental Figure S5a**). *Diptericin* levels showed high upregulation in all groups relative to controls, but this was only statistically significant in mTET2-R1261C and mTET2-R43G (**Supplemental Figure S5b**). *AttacinC* and *TotA* levels were also comparable to RNA-seq data (**Supplemental Figure S5c, d, Supplemental Table S3**).

### Fewer number of genes are dysregulated in mTET2-R43G and mTET2-R1261C mutants compared to mTET2-wt

**Figure 5.**
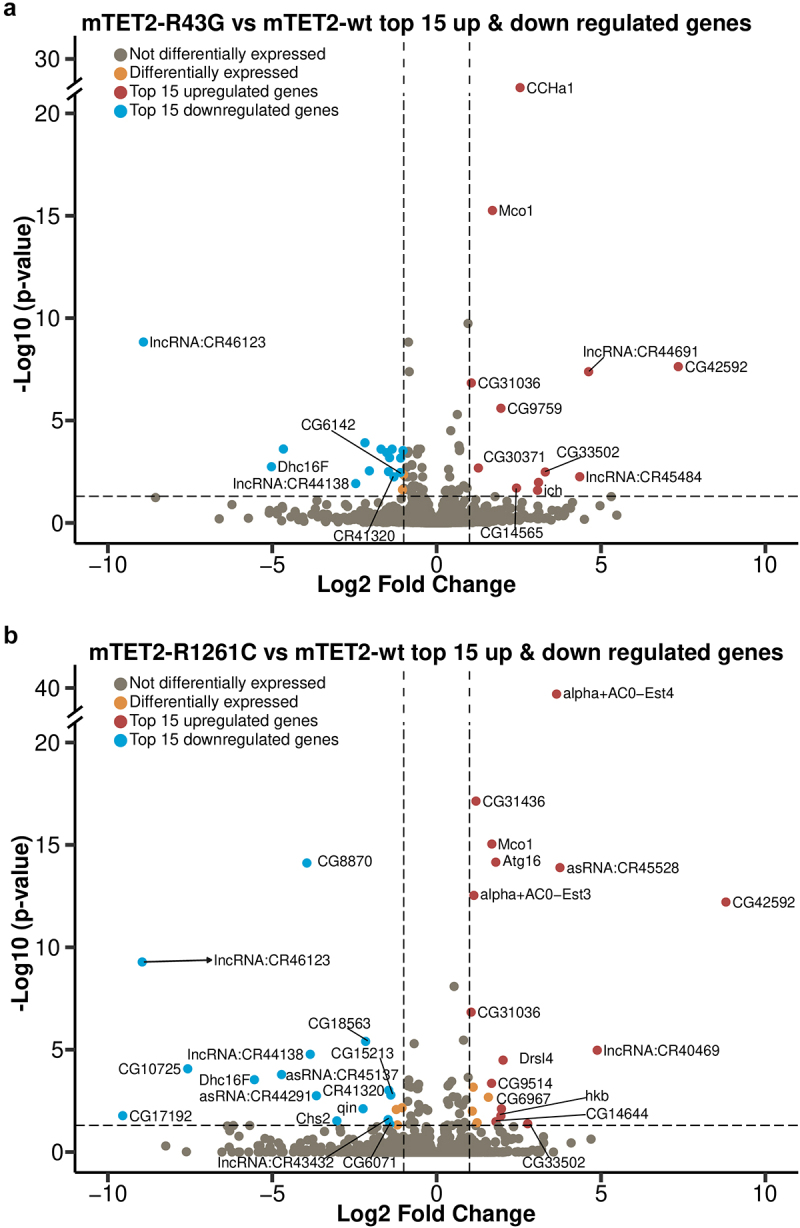
**Top 15 enriched genes in mTET2-R43G and mTET2-R1261C fly heads compared to mTET2-wt**. Volcano plots representing the most significantly up- or down-regulated genes. Several of these are commonly found in both mTET2-R43G and mTET-R1261C.

Next, we opted to investigate the DEGs that were identified when comparing both mTET2-R43G and mTET2-R1261C mutants to mTET2-wt. Several of these DEGs had unknown functions. One of the genes that was significantly downregulated in both mTET2-R43G and mTET2-R1261C mutants was *Dhc16F* (LogFC= −5.02 and −5.53, respectively) (Figure 5a,b). *Dhc16F* is expressed in scolopidial neurons that are important for mechanosensation and in other tissues such as spermatogonium, spermatozoon, and testis [30,31]. Furthermore, some long non-coding RNAs were also downregulated in both mTET2-R43G and mTET2-R1261C mutants including lncRNA: CR46123 (LogFC= −8.91 and −8.95, respectively) (Figure 5a,b, Supplemental Table S3). Another gene of interest was *CG33502*, which encodes a protein involved in iron ion binding activity and iron-sulphur cluster binding activity and is predicted to be involved in iron-sulphur cluster assembly (UniProt, Q8SY96). This gene was significantly upregulated in mTET2-R43G and mTET2-R1261C mutants (LogFC = 3.30 and 2.77, respectively). Another alarming finding is that the expression of two genes, *huckebein* (*hkb*) and *ichor* (*ich*) persisted in adulthood in the mutants although it typically ceases in pupal stages. Of note, *ich*, which is a zinc finger transcription factor implicated in DNA binding and activation of gene expression, was upregulated in mTET2-R43G compared to mTET2-wt (LogFC = 3.07) (Figure 5a). On the other hand, *hkb* was upregulated in mTET2-R1261C flies (LogFC = 1.95) (Figure 5b, Supplemental Table S3). *Hkb* is a zinc finger transcription factor expressed in patches within the embryonic neuroectoderm and a subset of neuroblasts where it is required for proper neuronal and glial cell specification and axon targeting. Another dysregulated gene of interest is autophagy-related 16 (*Atg16*) which is a protein coding gene involved in the maintenance of proper neuromuscular function and lifespan. *Atg16* was found to be upregulated in mTET2-R1261C mutants (LogFC = 1.79) (Figure 5b, Supplemental Table S3) and to a lesser degree in mTET2-R43G (LogFC = 0.82, p-adj = 0.01).

### Downregulation of atg16 and ich in mTET2-R43G expressing flies increases survival rate and rescues circadian phenotypes

**Figure 6.**
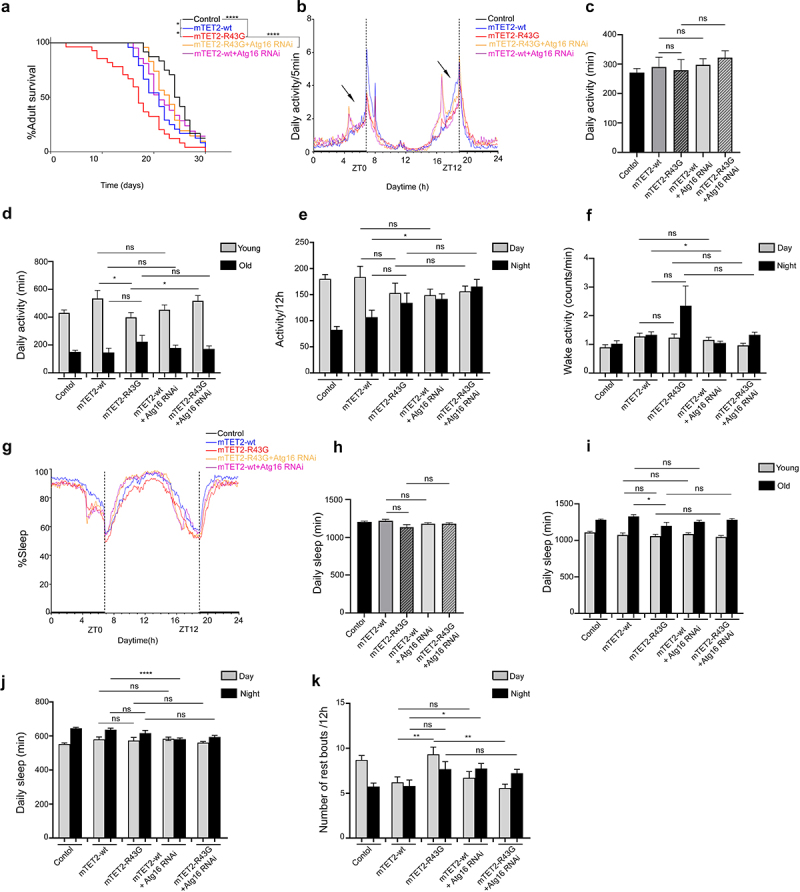
**Knocking down atg16 gene in mTET2-R43G expressing flies rescues the survival, altered activity, and circadian phenotypes**. (**a**) Kaplan-Meier survival curve of male flies expressing no transgene (*w*^*1118*^ control, *n* = 24), mTET2-wt (*n* = 24), mTET2-R43G (*n* = 28), mTET2-R43 G+atg16 RNAi (*n* = 24), or mTET2-wt+atg16 RNAi (*n* = 21) driven by dTet-Gal4. Statistical significance of the difference between survival curves was determined using the Mantel-Haenszel test. Survival of mTET2-R43 G+atg16 RNAi was significantly higher and almost similar to the *w*^*1118*^ control and mTET2-wt flies compared to mTET2-R43G flies (****p < 0.0001). (**b**) Locomotor activity graphs were analysed over 30 days. For each group, the locomotor activity levels of male flies were measured in 5-minute bins and then averaged to obtain a representative activity profile. *Drosophila melanogaster* generally exhibits two activity bouts: one centred around ZT0 (morning peak) and the second around ZT12 (evening peak). (**c**) Graph showing the average activity of the flies over 24 h intervals (Unpaired t-test; ns: *p* > 0.05). (**d**) the flies are subdivided into two groups in an age-dependent manner since locomotion is age-dependent: ‘young’ refers to 1–13 days old and ‘old’ refers to 14–30 days old. mTET2-R43G young flies are significantly less active (* *p* < 0.05), compared to mTET2-wt, whereas young mTET2-R43 G+atg16 RNAi expressing flies are more active compared to mTET2-R43G young flies (* *p* < 0.05). (**e**) Graph showing the average locomotor activity over 12 h intervals (day: light on, night: light off). During the night, mTET2-wt+atg16 RNAi flies are more active compared to mTET2-wt flies (Unpaired t-test; * *p* < 0.05). (**f**) Graph illustrating the wake activity counts per minute over 12 h intervals. The wake activity is a measure of the activity rate when the flies are awake. mTET2-wt+atg16 RNAi flies demonstrate higher wake activity at night compared to mTET2-wt flies (Unpaired t-test; * *p* < 0.05). (**g**) Graph illustrating the percent of the time that flies spend sleeping over several days. For each group, the percent of flies sleeping was measured in 5-minute bins and then averaged to obtain a representative sleep profile. ZT0 indicates the morning peak and ZT12 the evening peak. (**h**) Graph showing the average sleep of the flies over 24 h intervals. (Unpaired t-test; ns: *p* > 0.05). (**i**) the flies are subdivided into two age groups considering that sleep is also age-dependent: ‘young:’ 1-13 days old and ‘old:’ 14–30 days old (Unpaired t-test; * *p* < 0.05, ns: *p* > 0.05). (**j**) Graph showing the average of daily sleep minutes for all flies over 12 h intervals over 30 days. During the night, mTET2-wt+atg16 RNAi flies showed a significant decrease in sleep time compared to mTET2-wt flies. (Unpaired t-test, ****: *p* < 0.0001). (**k**) Graph showing the average number of rest bouts for all flies in one group for 12 h intervals over 30 days. During the day, mTET2-R43G flies showed significantly more rest bouts compared to mTET2-wt flies (Unpaired t-test, **: *p* < 0.005). During the day, mTET2-R43 G+atg16 RNAi flies showed significantly fewer rest bouts compared to mTET2-R43G flies (Unpaired t-test; **p < 0.005). mTET2-wt+atg16 RNAi flies showed significantly more rest bouts compared to mTET2-wt flies (Unpaired t-test; * *p* < 0.05).

**Figure 7.**
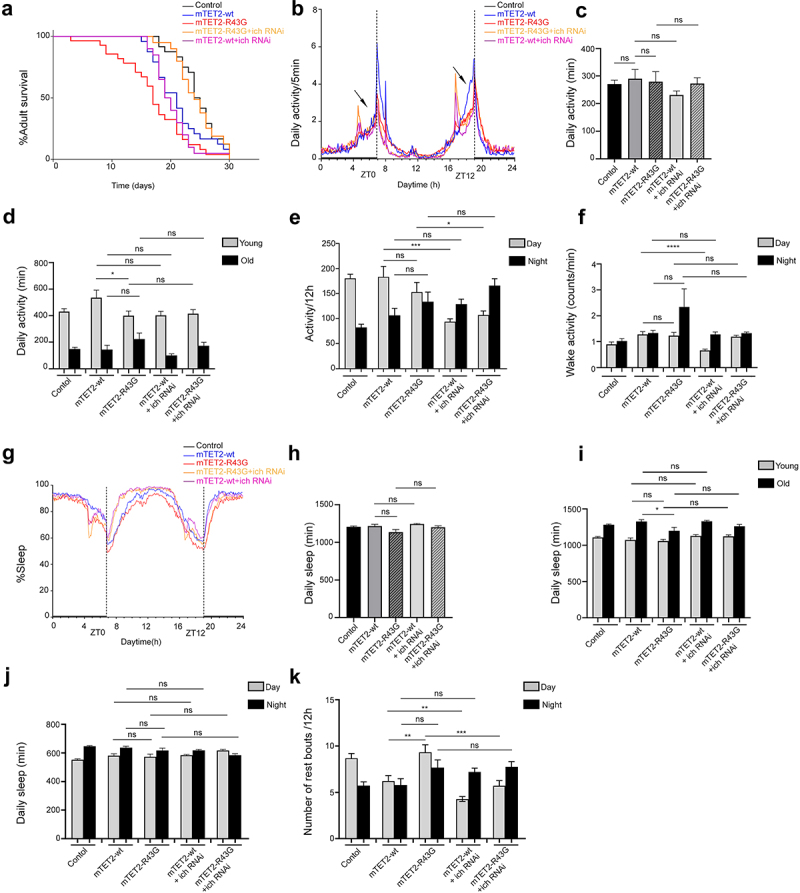
**Knocking down ich gene in mTET2-R43G expressing flies impacts the survival, activity, and circadian phenotypes. (a)** Kaplan-Meier survival curve of male flies expressing no transgene (*w*^*1118*^ control, *n* = 24), mTET2-wt (*n* = 24), mTET2-R43G (*n* = 28), mTET2-R43 G+ich RNAi (*n* = 26), mTET2-wt+ich RNAi (*n* = 20) driven by dTet-Gal4. Statistical significance of the difference between survival curves was determined using the Mantel-Haenszel test. Survival of mTET2-R43 G+ich RNAi was significantly higher and similar to the control compared to mTET2-R43G flies (***p < 0.0001). (**b**) Locomotor activity graphs were analysed over 30 days. For each group, the locomotor activity levels were measured in 5-minute bins and then averaged to obtain a representative activity profile. The activity bout centred around ZT0 represents the morning peak while ZT12 represents the evening peak. mTET2-R43 G+ich RNAi flies exhibit abnormal hyperactivity relative to all other groups. (**c**) Graph showing the average activity of all flies over 24 h intervals (Unpaired t-test; ns: *p* > 0.05). mTET2-R43 G+ich RNAi expressing flies are significantly more active than mTET2-R43G flies (* *p* < 0.05). (**d**) Flies were subdivided into two groups by age, with ‘young’ representing 1–13 days old and ‘old’ representing 14–30 days old. mTET2-R43G young flies are significantly less active than mTET2-wt (*p < 0.05). (**e**) Graph showing the average locomotor activity over 12 h intervals (day: light on, night: light off). During the day, mTET2-wt+ich RNAi flies are significantly less active compared to mTET2-wt flies (Unpaired t-test; *** *p* < 0.0005). mTET2-R43 G+ich RNAi expressing flies display significantly higher activity during the day and night compared to mTET2-R43G flies (Unpaired t-test; * *p* < 0.05, ** *p* < 0.005). (**f**) Graph illustrating the wake activity counts per min over 12 h intervals. The wake activity is a measure of the activity rate when flies are awake. mTET2-wt +ich RNAi flies demonstrate significantly lower wake activity during the day in contrast to mTET2-wt flies (Unpaired t-test, ****: *p* < 0.0001). (**g**) Graph illustrating the proportion of time that flies spend sleeping over several days. The percent of flies sleeping was measured in 5-minute bins and then averaged to obtain a representative sleep profile. ZT0 indicates the morning peak and ZT12 indicates the evening peak. (**h**) Graph showing the average sleep of the flies over 24 h intervals. (Unpaired t-test; ns: *p* > 0.05). (**i**) Flies were subdivided into two groups by age, with ‘young’ representing 1–13 days old and ‘old’ representing 14–30 days old. (Unpaired t-test; * *p* < 0.05, ns: *p* > 0.05). (**j**) Graph showing the average of daily sleep minutes for all flies over 12 h interval over 30 days (Unpaired t-test, ns: *p* > 0.05). (**k**) Graph showing the average number of rest bouts for all flies in one group for 12 h intervals over 30 days. During the day, mTET2-R43G flies showed significantly more rest bouts compared to mTET2-wt flies (Unpaired t-test, **: *p* < 0.005). mTET2-R43 G+Iich RNAi flies showed significantly fewer rest bouts during the day compared to mTET2-R43G flies (Unpaired t-test; ***p < 0.0005). Similarly, during the day, the number of rest bouts was significantly lower in mTET2-wt+ich RNAi expressing flies compared to mTET2-wt flies (Unpaired t-test; **p < 0.005).

We aimed to understand whether there was any direct correlation between some of the dysregulated genes and the phenotypes observed upon expression of different mTET2 mutants. For this, we focused on *atg16* and *ich*, which were both upregulated in mTET2-R43G mutants, although upregulation in mTET2-R43G was not was not as explicit as in mTET2-R1261C. Considering that circadian behaviour defects could also be correlated with the dysregulated genes upon mutant mTET2 expression, we therefore knocked down selective candidate genes by RNA interference and assessed whether this could rescue the phenotypes induced by the mTET2-R43G mutation by examining survival and behaviour phenotypes specifically. Consistent with previous experiments, the mTET2-R43G mutants showed an overall decrease in survival compared to mTET2-wt flies (Figure 6a). Notably, mTET2-R43G mutants with *atg16* or *ich* knockdown displayed a prolonged lifespan (Median survival of 23 and 24 days, respectively) compared to mTET2-R43G mutants (Median survival of 17 days) (Figures 6a, 7a). Although survival was reduced in mTET2-wt flies with respect to the controls (Median survival of 21 and 24.5 days, respectively), mTET2-wt with *atg16* or *ich* knockdown flies did not exhibit any significant enhancements in lifespan (Median survival of 21, *P* = 0.3405 and 19.5 days, *P* = 0.465 respectively) (Figures 6a, 7a). These findings imply that knocking down both *atg16* and *ich* rescues and prolongs the survival of mTET2-R34G mutants specifically, but not mTET2-wt flies. We further assessed the impact of knocking down *atg16 or ich* alone on the adult’s lifespan. Knocking down *atg16* or *ich* affected the survival of the adult life span with a median survival of 20 and 13 days respectively **(Supplemental Figure S3)**. Despite this effect, knocking down both genes in mTET2-R43G background improved survival of these flies significantly (Figures 6, 7 **and Supplemental Figure S3)**. Notably, the decrease in activity during the anticipation phase before evening (ZT12) was not rescued with either *atg16* or *ich* knockdown in mTET2-R43G flies (Figures 6b, 7b). The mean daily activity over 24 h or 12 h and the wake activity did not significantly change upon *atg16* or *ich* knockdown in mTET2-R43G relative to mTET2-wt flies (Figures 6(c,e,f) and Figure 7(c,e,f). Compared to mTET2-R43G mutants, young mTET2-R43G flies with *atg16* knockdown exhibited a significant increase in average daily activity which was also confirmed by the actogram plotted for these flies (Figure 6d, Supplemental Figure S4). Moreover, the reduced average daily sleep in old mTET2-R34G mutants was not enhanced in old mTET2-R43G with *atg16* knockdown flies (Figure 6i). In addition, young or old flies expressing mTET2-R43G with *ich* knockdown did not show any significant changes in average daily activity or sleep (Figure 7d,i). These findings indicate that knocking down *atg16* but not *ich* fully rescued the daily locomotor defects in mTET2-R43G flies at a young age. On the other hand, the average daily sleep over 24 h or 12 h did not show significant changes after knocking down *atg16* or *ich in* mTET2-R43G flies compared to mTET2-wt flies (Figure 6(g,h,j) and Figure 7(g,h,j)). Interestingly, knocking down *atg16* and *ich* in both mTET2-wt and mTET2-R43G flies led to a significant reduction in the number of sleep bouts during light (day) phases (Figures 6k, 7k). Taken together, downregulating *atg16* and *ich* in mTET2-R43G mutants rescued the survival and some of the behavioural phenotypes, suggesting the importance of these genes for proper locomotor and circadian behaviour as well as adult fly longevity.

### Downregulation of humoral immune pathways restores haemocyte homoeostasis

**Figure 8.**
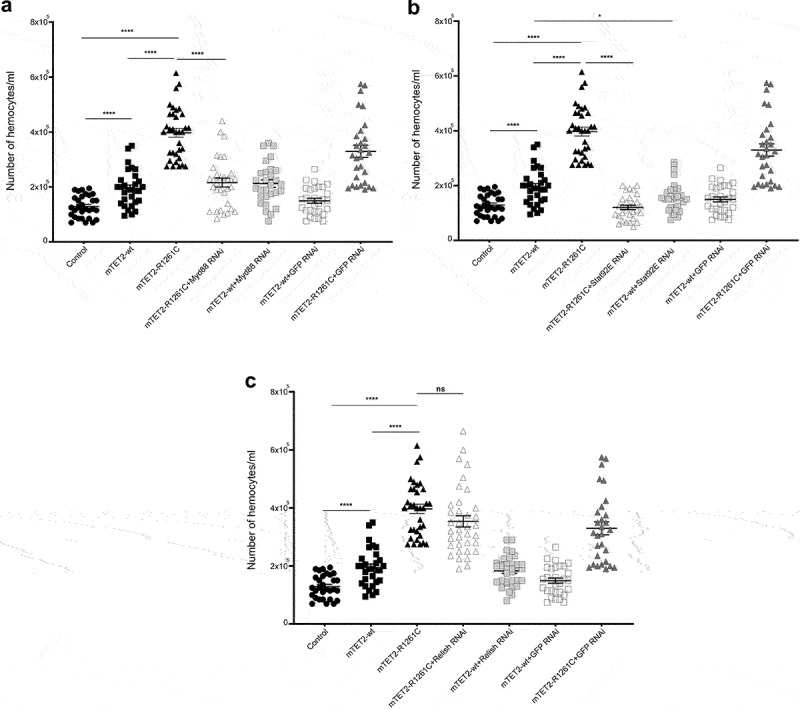
**Expression of Myd88 RNAi and Stat92E RNAi in mTET2-R1261C expressing larvae rescues the increase in the number of haemocytes**. Graphs showing the number of haemocytes in third instar larvae expressing either no transgene (*w*^*1118*^ control), mTET2-wt, or mTET2-R1261C, mTET2-R1261C + Myd88 RNAi, mTET2-wt + Myd88 RNAi, mTET2-R1261C + Stat92E RNAi, mTET2-wt + Stat92E RNAi, mTET2-R1261C + Relish RNAi, mTET2-wt +Relish RNAi, mTET2-R1261C + GFP RNAi, or mTET2-wt + GFP RNAi driven by dTet-Gal4 (n ≅ 30). (**a**) mTET2-R1261C + Myd88 RNAi expressing larvae show a significant decrease in the number of haemocytes compared to mTET2-R1261C larvae (Unpaired t-test; ****p < 0.0001). (**b**) Number of haemocytes in mTET2-R1261C + Stat92E RNAi expressing larvae was significantly reduced compared to mTET2-R1261C larvae (Unpaired t-test; ****p < 0.0001). mTET2-wt + Stat92E RNAi expressing larvae showed a significant decrease in haemocytes number compared to mTET2-wt larvae (Unpaired t-test; * *p* < 0.05). (**c**) mTET2-R1261C + Relish RNAi expressing larvae showed no significant difference in the number of haemocytes compared to mTET2-R1261C larvae (Unpaired t-test; ns: *p* > 0.05).

Since expression of the R1261C mutant prevalent in leukaemia caused a dramatic increase in haemocyte numbers and many innate immune genes were upregulated at the transcriptional level, we tested whether downregulation of key innate effector genes could rescue these phenotypes in mTET2-R1261C flies. For this, we expressed mTET2-R1261C in dTet-expressing cells with either Myd88, Relish, or Stat92E RNAi targeting the three main pathways that play a role in innate immunity: the Toll pathway, the immune deficiency pathway (IMD), and the JAK-STAT pathway. For a negative control, we further expressed GFP RNAi in mTET2-wt and mTET2-R1261C flies. The haemocyte number was quantified in third instar larval progeny and tested for phenotypic rescue. The number of haemocytes was drastically reduced in mTET2-R1261C with Myd88-RNAi (Mean = 2.15×10^5^/mL) (Figure 8a) and Stat92E RNAi lines (Mean = 1.2×10^5^/mL) (Figure 8b) when compared to mTET2-R1261C mutants (Mean = 3.97×10^5^/mL). mTET2-R1261C+Relish RNAi larvae did not show a significant variation in haemocyte number (Mean = 3.53×10^5^/mL) in contrast to mTET2-R1261C larvae (Mean = 3.97×105/mL) (Figure 8c). Interestingly, mTET2-wt+Stat92E RNAi larvae exhibited a remarkable decrease in haemocyte number (Mean = 1.58 × 10^5^/mL) with respect to mTET2-wt larvae (Mean = 1.94 × 10^5^/mL) (Figure 8b). Considering these findings, we speculate that mTET2-R1261C may play a role in altering haemocyte homoeostasis and that targeting the Toll and JAK-STAT humoral pathways contributes to restoring normal haemocyte numbers in mTET2-R1261C mutants.

## Discussion

Epigenetic balance is maintained by a network of proteins and any departure from this homoeostasis, whether resulting in a permissive or resistant state, can lead to the activation of oncogenic programs [32]. TET2 was found to be mutated in AML, MDS, and CMML [29,33–35]. Most of these mutations resulted in the loss of catalytic activity of TET2, therefore pointing towards the importance of its catalytic function [36,37]. In this study, we developed a *Drosophila* model to understand the impact of expression of wild type mammalian TET2 as well as a mutation Arginine 43 to Glycine (R43G) that was identified in DIPG, a brainstem tumour that is highly aggressive and difficult to treat surgically [38]. A total of 69 TET2 mutations identified in CNS tumours are currently documented in *cBioPortal*. These include 12 missense mutations (putative driver mutations), 50 missense mutations with unknown significance, 5 truncating mutations (putative driver mutations), and 2 splice mutations (putative driver mutations). The percentage of samples with somatic mutations in TET2 as per *cBioPortal* is 1.1%. Of note, the R43G mutation was not found to be amongst the 69 TET2 mutations in *cBioPortal* indicating that substitution at this residue was first reported in our previous exome sequencing data. By using bioinformatics prediction tools, this mutation showed an intermediate biological effect and was therefore a good candidate for our *Drosophila* model. In addition, there are other missense somatic mutations that are present in glioma in TET2 outside the catalytic domain. We therefore also included the Arginine 1261 Cysteine (R1261C) catalytically inactive mutant that is commonly reported in AML patients [39]. Notably, this residue was also found to be mutated in a total of eight glioma samples, seven of which had the R1261C mutation and one had the Arginine 1261 Histidine R1261H mutation (*cBioPortal*). We first wanted to express wild type and mutant mTET2 in cells that express dTet in flies. This includes expression in the larval and adult brains, in both neural and glial cell populations, as well as the larval fat body, wing discs, and eye discs [24]. We verified the expression of transgenes and assessed the status of its catalytic activity, with a resulting change on both RNA and DNA modifications that TET is known to catalyse. Although dTet is capable of oxidizing 5mrC, mammalian TET proteins have been shown to perform this function *in vitro* only [40,41]. Our findings point towards the ability of mTET2 to act on 5mrC *in vivo* with clear reduction in 5hmC levels in adult brains of the catalytically inactive mutant mTET2-R1261C.

The expression of mTET2-wt, mTET2-R1261C, and mTET2-R43G in flies also led to gaps in the thorax and abdomen. This cleft phenotype has been described extensively in many studies which addressed the involvement of the JNK pathway in this critical thorax closure process [42–44]. However, recent studies with mutants that are unrelated to the JNK pathway have also reported this phenotype. For instance, another protein that is involved in epigenetic regulation is Alpha-Thalassaemia and mental retardation X-linked syndrome (ATRX) and it has been linked to thorax closure via its effect on expression of genes required for this process [45]. On the other hand, Activating transcription factor 3 (atf3) has been shown to play a crucial role in abdomen closure through its ability to interact with Jun and Fos from the JNK pathway [46]. This hints at the idea that genes that are involved in morphogenesis are more extensive than initially thought. Furthermore, the expression of the mTET2 transgenes also led to a structural malformation in male genitalia. Interestingly, this phenotype is also observed in JNK pathway dysregulation, but it has only been described in a few previous studies [47,48]. Although these phenotypes may seem distant from the mammalian system, the molecular players and signalling pathways are conserved as the JNK pathway is known to be dysregulated in cancers. In our study, we hypothesize that excessive inhibition of the JNK pathway caused the observed phenotypes because of defective or incomplete migration of epithelial cells. Surprisingly, Puckered (Puc), the sole *Drosophila* Jun N-terminal kinase (JNK)-specific MAPK phosphatase (MKP) was not among the differentially regulated genes in the RNA-seq that was performed on adult heads. Puckered is expressed in moderate levels in the adult brain but this has not been studied in detail and there is a lack of information on the role of the JNK pathway in the fly brain. Although none of the DEGs that were identified in the mutants were directly related to the JNK pathway, this does not exclude the possibility that some of these genes may be connected to the JNK pathway or that regulation may occur at the post-transcriptional level.

We also observed melanotic or necrotic abdominal masses in a subset of adult flies expressing wild type or mutant mTET2. The appearance of these masses in flies has typically been attributed to a hyperactive immune system. Melanization is a protective process that is activated in response to an immune stressor by a specific class of haemocytes (blood cells), the crystal cells. Melanin accumulation at a site of injury can lead to immobilization of pathogens and initiate the wound healing process [49]. In addition, the melanization process also produces free radicals that can kill pathogens [50]. The peptides that are secreted upon activation of the three immune pathways, the Toll pathway, IMD pathway, and JAK-STAT pathway, were found to be significantly upregulated in the mTET2-wt, mTET2-R1261C, and mTET2-R43G flies. Most molecules that are involved in these pathways have conserved homologs in the mammalian immune system. The IMD pathway is homologous to the TNF-α pathway while the Toll pathway resembles the NF-κB pathway [51]. Previous evidence indicated a correlation between AMPs overexpression and neurodegeneration where flies with high AMP brain levels demonstrated reduced lifespan and locomotor deficit [52]. In their study, blocking the Toll pathway was effective in reducing neurodegeneration and restoring survival in these flies. As such, in our model, the mTET2 mutation may act as a proactive signal that is crucial for boosting the initiation of innate immune response. Although the status of IMD and Toll pathways in glioma is not well characterized, our data showed that AMP genes related to these signalling pathways were remarkably upregulated in mTET2-R43G and mTET2-R1261C brains. This indicates that Toll and IMD pathways might be implicated in the brain humoral immune response in mTET2 mutants. A recent study demonstrated the involvement of Toll pathway in the immune reaction of the nervous system. In the latter study, spätzle, which directly activates Toll pathway, was proposed to play a role in suppressing gliomagenesis and glioblastoma progression [53]. Moreover, the JAK-STAT pathway has also been reported to be involved in tumorigenesis and the formation of melanotic tumours [54,55]. *TotA* and *TotB*, which are transcribed in response to activation of the JAK-STAT pathway, were upregulated in wild-type mTET2 heads, indicating that TET2 can interact with – and perhaps activate – these immunity-related signalling pathways. Future work should aim to identify IMD and Toll targets in glial cells to better understand the mechanisms of AMP dysregulation in the brain by both dTet and the exogenous dTet expression of mTET2. These AMPs could be imported from the haemolymph into the brain or secreted by haemocytes or glial cells in the brain upon mTET2 expression. It is therefore critical to assess whether the immunity triggered in the brain is brain cell-specific or is induced by the fat body initially upon mTET2 expression. In mTET2-R1261C mutants, the immune response was characterized by melanotic masses in adult flies and increased haemocyte number in larvae. A possible explanation for the increased haemocyte count could be a dysregulation in the expression of genes implicated in haemocyte proliferation, resulting in increased circulating haemocytes throughout larval development. Blocking either Myd88/Toll or Stat92E/JAK-STAT pathways reduced the increase haemocyte numbers induced upon expression of mTET2-R126C. This has been consistently documented in previous leukaemia fly models where oncogene expression in flies induced haemocyte expansion and formation of melanotic tumours [56,57]. One example is mutations in RAS inducing an increase in the number of circulating haemocyte and dysregulation in Toll and IMD pathway in response to oncogenic distress [58]. Accordingly, the observed haemocyte increase is more likely to be due to disruption in the proliferation of circulating haemocytes. Additionally, JAK-STAT signalling is known to regulate *Drosophila* haematopoiesis by regulating haemocyte proliferation or differentiation [59]. It has been further demonstrated that in response to tissue damage, the JAK-STAT pathway promotes haemocyte proliferation [60] which is in line with our findings where JAK-STAT components were upregulated in mTET2 mutants. Since Relish knockdown in mTET2-R1261C mutants did not rescue the increase in haemocyte numbers, we hypothesize that the IMD pathway might not be directly involved in this process. Moreover, many DEGs that were found in the transcriptome analysis in mTET2 expressing flies relative to controls were indirectly related to the immune system. Genes coding for serine proteases that are important in melanization reaction within haemocytes were dysregulated in mTET2-wt, mTET2-R1261C, and mTET2-R43G. mTET2 did not only affect haemocyte haemostasis but also glial cell numbers in brain. Although there were no obvious transcriptomic changes in classical genes involved in the proliferation or differentiation of glial/neuronal cells in the brain, it is still possible that some of the identified DEGs are involved in this process. Expression of *huckebein* (*hkb*), a zinc finger transcription factor required for glial development and differentiation [61], persisted in flies expressing mTET2-R43G and mTET2-R1261C in adulthood and it has been shown that the upregulation of *hkb* is sufficient to increase the number of glia in the fly brain [62]. It is unclear whether this expansion in glia is due to increased proliferation or a shift in differentiation that favours glia versus neurons.

Other candidates that were among the DEGs in mutant TET2 flies and have been reported to be generally dysregulated in cancers include MMPs and other enzymes. Genes of the Neprilysin family of MMPs were upregulated in mTET2 mutants compared to controls. A previous study on a fly glioma model reported an upregulation in MMPs, which is thought to be indicative of invasive ability [63]. Concomitant with the glial expansion, flies expressing mTET2-R43G exhibited reduced lifespan and disruption in circadian behaviour compared to mTET2-wt. Although it is not clear how glial cells might contribute to mTET2-R43G related changes in circadian behaviour, previous studies illustrated the role of adult glial cells in modulating the circadian neuronal network and behaviour in *Drosophila* [64]. A recent study reported a disruption in the locomotor circadian rhythm upon overexpression of an astrocyte-derived neurotrophic factor in astrocytes which modulated the activity of the clock neurons and thus circadian behavioural changes [65]. Moreover, the circadian behaviour normally tends to diminish as the flies age which dampens cellular homoeostasis and affects locomotor behaviour [66]. In this context, our data indicates deficits in activity and sleep in the mTET2-R43G mutation in an age-specific manner. In *Drosophila* brain, the circadian clocks are organized in several neuronal clusters that communicate with each other through chemical and synaptic transmission controlling the morning and evening circadian events [67,68]. Any interference in these synaptic connections may lead to undesired disruption in the locomotor activity which is a representation of the circadian clock activity. Thus, a normal circadian behaviour is established by functional neuron-behaviour associations. Consistent with our findings, *Drosophila* Tet has previously shown to have a role in normal behaviour and neuronal network by regulating the development of neurons expressing the pigment dispersing factor (PDF), a key neurotransmitter in the circadian clock that is required to maintain bimodal circadian rhythms [64]. Furthermore, the increase in the rest bouts noticed in mTET2-R43G expressing flies may indicate that these flies display disrupted sleep phases. One of the surprising findings in the study is that mTET2-wt expression itself resulted in increased glial cell population in adults and displayed morphological defects and phenotypic increase in haemocytes, indicating that ectopic expression of TET2 itself can cause dramatic changes albeit to a lesser extent than the mutants. This also suggests that the specific level of TET expression in flies is crucial for proper development, glial haemostasis, behaviour, and circadian rhythm. In fact, previous studies reported overexpression of TET2 in colorectal carcinoma, glioblastoma, melanoma cells, and chronic lymphocytic leukaemia patients [69,70]. It will be of interest to determine which glial cell population is affected in TET2-R43G mutant brains and whether there is a direct link with proliferation and circadian defects. Additionally, overexpression of *Drosophila* Tet (dTet) was detrimental on the survival of the flies during development and adulthood indicating that dTet overexpression could be less tolerated compared to the m TET2 overexpression. This could be due to the difference in the substrate specificity between mammalian TETs and dTet as demonstrated by previous studies that mammalian TETs demethylate 5mC on DNA compared to dTet that demethylates 5mC on RNA and 6 mA on DNA [71,72].

Our transcriptomic analysis and knockdown studies also revealed a role for autophagy-related genes. These genes display great potential as a novel therapeutic target and hence it will be of importance to delineate the extent to which they are correlated with cell survival in TET2 tumour models. In addition, deciphering the mechanisms involved in the immune response to tumours is essential to our understanding of immune recognition and cancer progression. Therefore, the current TET2 *Drosophila* model could be a powerful tool to identify new genes and pathways involved in tumorigenesis as a result of TET2 dysregulation. Our results indicate that non-catalytic TET2 similar to catalytic mutant TET2 is important in regulating essential mechanisms pertaining to fly survival, locomotion and circadian rhythms that warrants further assessments to better understand the role of TET2 in development and cancer.

## Supplementary Material

Supplemental Material

## Data Availability

RNA-seq datasets for Drosophila samples have been deposited in the Gene Expression Omnibus (GEO) under accession number GSE213118.

## Highlights

- mTET2-R43G, mTET2-R1261C, and wild type mTET2 expression leads to phenotypic defects in adult fly thorax, male genitalia, and formation of abdominal melanotic masses.
- mTET2-R43G mutants display behavioural and lifespan defects, whereas the mTET2-R1261C mutation alters the larval haemocyte haemostasis.
- Transcriptomic analysis showed an upregulation in several immune-related genes in mutant and wild type mTET2 flies with few genes differentially expressed in mTET2 mutant flies.
- Knockdown of *atg16* and *ich* genes in mTET2-R43G flies rescues survival and behavioural phenotypes.
- Silencing humoral immune pathway genes *Myd88* or *STAT92E* in mTET2-R1261C mutants restores larval haemocyte homoeostasis.
