# Supplementary material for: Phenotypic and transcriptomic impact of expressing mammalian TET2 in the Drosophila melanogaster model
Source: Epigenetics. 2023 Mar 29;18(1):2192375. doi: 10.1080/15592294.2023.2192375 (PMC10072067; doi:10.1080/15592294.2023.2192375)
Supplement: Supplemental Material [file KEPI_A_2192375_SM8246.zip › Supplementary files/Supplementary figures and tables caption.docx]

**Supplementary Figure 1:**  **Schematic illustration of the crosses schemes used for transcriptomic analysis**. UAS/GAL4 system is used to express the mTET2 transgenes in dTet expressing tissues using the dTet Gal4 promotor. To obtain this expression, the fly system must include the transcriptional activator protein; GAL4 that is under the control of a tissue-specific promotor, and the gene of interest coupled to the UAS (Upstream Activating Sequence) which is a sequence recognized by GAL4. When GAL4 is expressed, it binds UAS to drive the transcription of gene of interest in a tissue-specific manner. The UAS/GAL4 system can be used in the same manner to knockdown a gene using RNA interference; RNAi.

**Supplementary Figure 2: Survival defects exhibited by TET expressing flies.** Flies overexpressing dTet show significant survival defects with low percent of pupae eclosing into adults. Overexpressing mTET2-wt with dTet RNAi displays a slight reduction in the number of eclosed pupae. (n=3, 6-20 larvae per group; *p < 0.05, ****p < 0.0001).

**Supplementary Figure 3: Survival analysis in adult transgenic and RNAi flies**. Kaplan-Meier survival curve of male flies expressing w1118 control (n=24); mTET2-wt (n=24); mTET2-R43G (n=28); dTet (n=21); mTET2-wt+dTet RNAi (n=29); mTET2-R43G+Atg16 RNAi (n=24); mTET2-wt+Atg16 RNAi (n=21); mTET2-R43G+ich RNAi (n=26), mTET2-wt+ich RNAi (n=20); Atg16 RNAi (n=19); ich RNAi (n=25); dTet-Gal4 (n=14). All mTET2 transgenes and RNAi lines are driven by dTet-Gal4. Statistical significance of the difference between survival curves was determined using the Mantel-Haenszel test. dTet-Gal4 expression did not have an impact on the survival of flies whereas the survival of dTet overexpressing flies was significantly reduced compared to mTET2-wt flies **(A)**. More severe life span reduction was observed in adult mTET2 flies with dTet RNAi compared to mTET2 expression alone **(B)**. Knocking down Atg16 and ich reduced the survival of the adult life span **(C and D respectively)**. (*p < 0.05, **p < 0.01, ***p < 0.001 and ****p < 0.0001).

**Supplementary Figure 4: Average locomotor actograms illustrating the activity of flies over 30 days at 25°C.** The actograms are plotted for each of the following fly genotypes: Control (*w^1118^*), mTET2-wt, mTET2-R43G, mTET2-R1261C, R43G+Atg16 RNAi, mTET2-wt+Atg16 RNAi, R43G+ich RNAi, and mTET2-wt+ich RNAi. Flies were exposed to 12:12 h light/dark cycles for 30 days at 25°C. Note that, the graphs do not include the dTet-Gal4 driver added to the text underneath each graph. The flies show bimodal activity profiles with morning (ZT0) and evening (ZT12) peaks.

**Supplementary Figure 5: Immune-related genes are upregulated in mTET2 flies relative to controls.** (**A-D**) qRT-PCR on adult heads for Drosomycin, Diptericin, AttacinC, and TotA (*, p<0.05, **, p<0.01, ****, p<0.0001, n=2, 30 flies per group).

**Supplementary Table 1:** Sequences of the primers used for qRT-PCR: *Rp49, Diptericin, Drosomycin, AttacinC,* and *TotA*.

**Supplementary Table 2.** **Table summarizing the transgenic and RNAi fly lines used.**

**Supplementary Table 3:** The differentially expressed genes (DEGs) in mTET2-wt relative to control, and mTET2 mutants relative to control or to mTET2-wt.
