# Supplementary figures and images for: Phenotypic and transcriptomic impact of expressing mammalian TET2 in the Drosophila melanogaster model
Source: Epigenetics. 2023 Mar 29;18(1):2192375. doi: 10.1080/15592294.2023.2192375 (PMC10072067; doi:10.1080/15592294.2023.2192375)

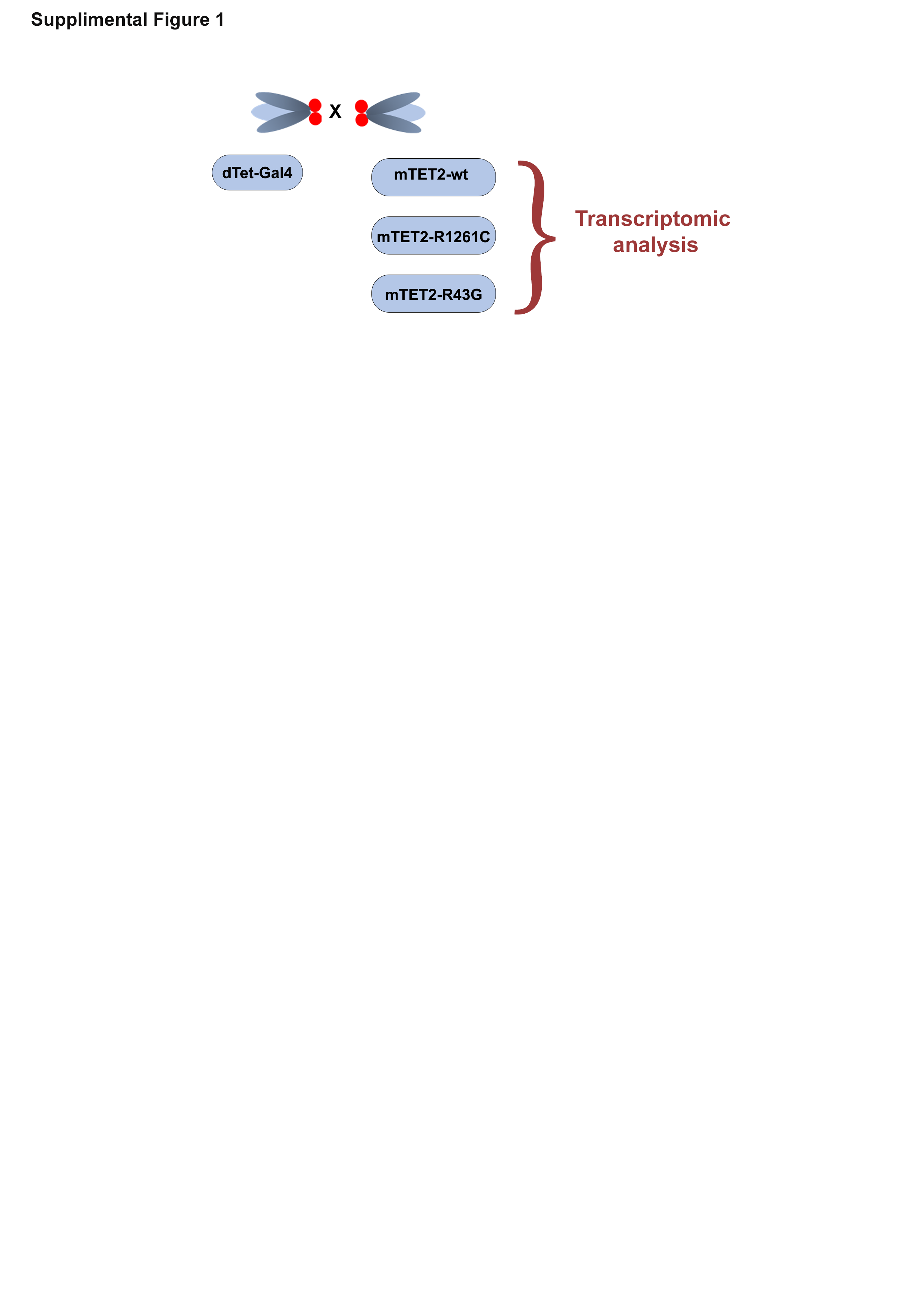

Supplement: Supplemental Material [file KEPI_A_2192375_SM8246.zip › Supplementary files/Supplemental Fig 1 .tif]

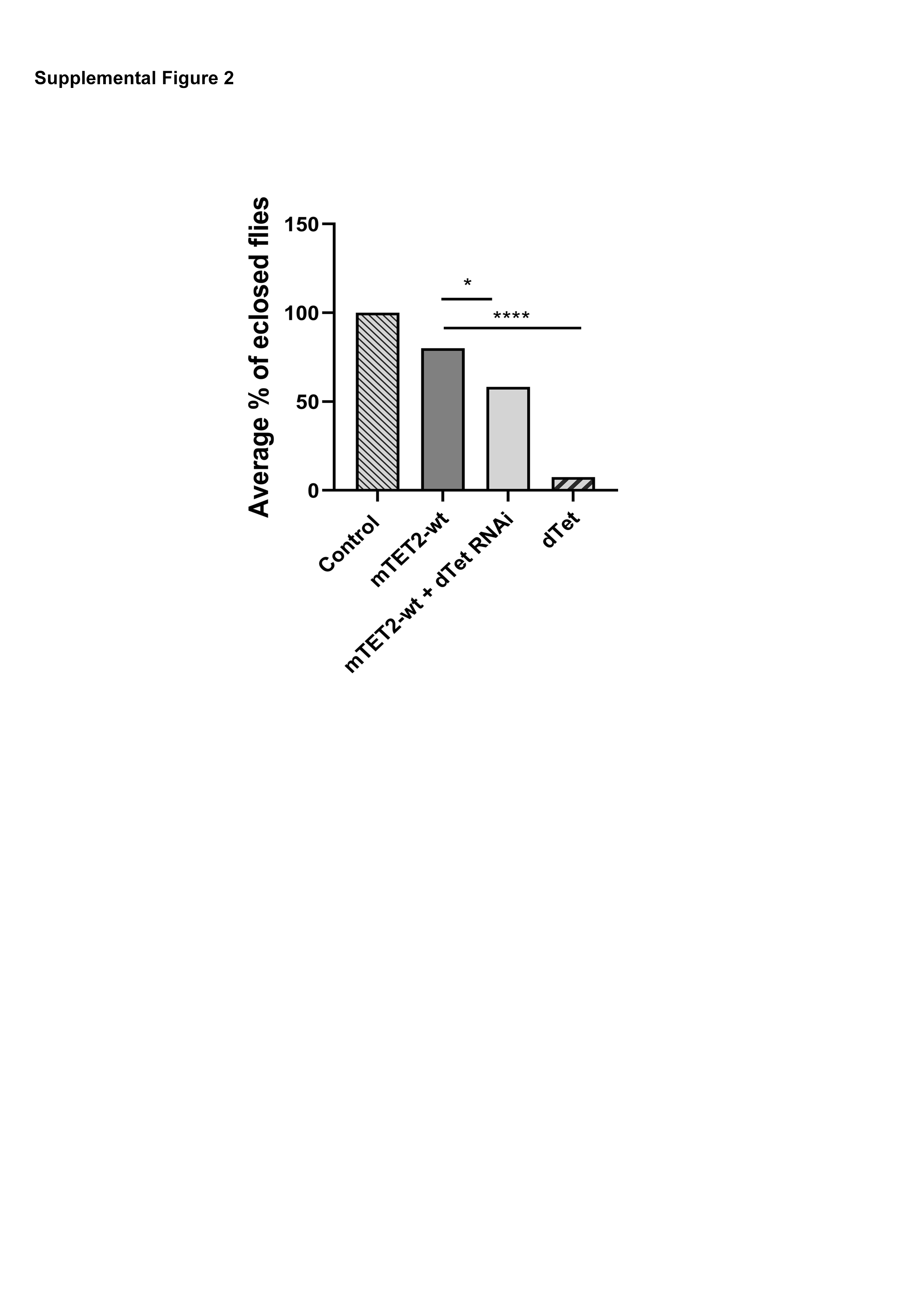

Supplement: Supplemental Material [file KEPI_A_2192375_SM8246.zip › Supplementary files/Supplemental Figure 2.tif]

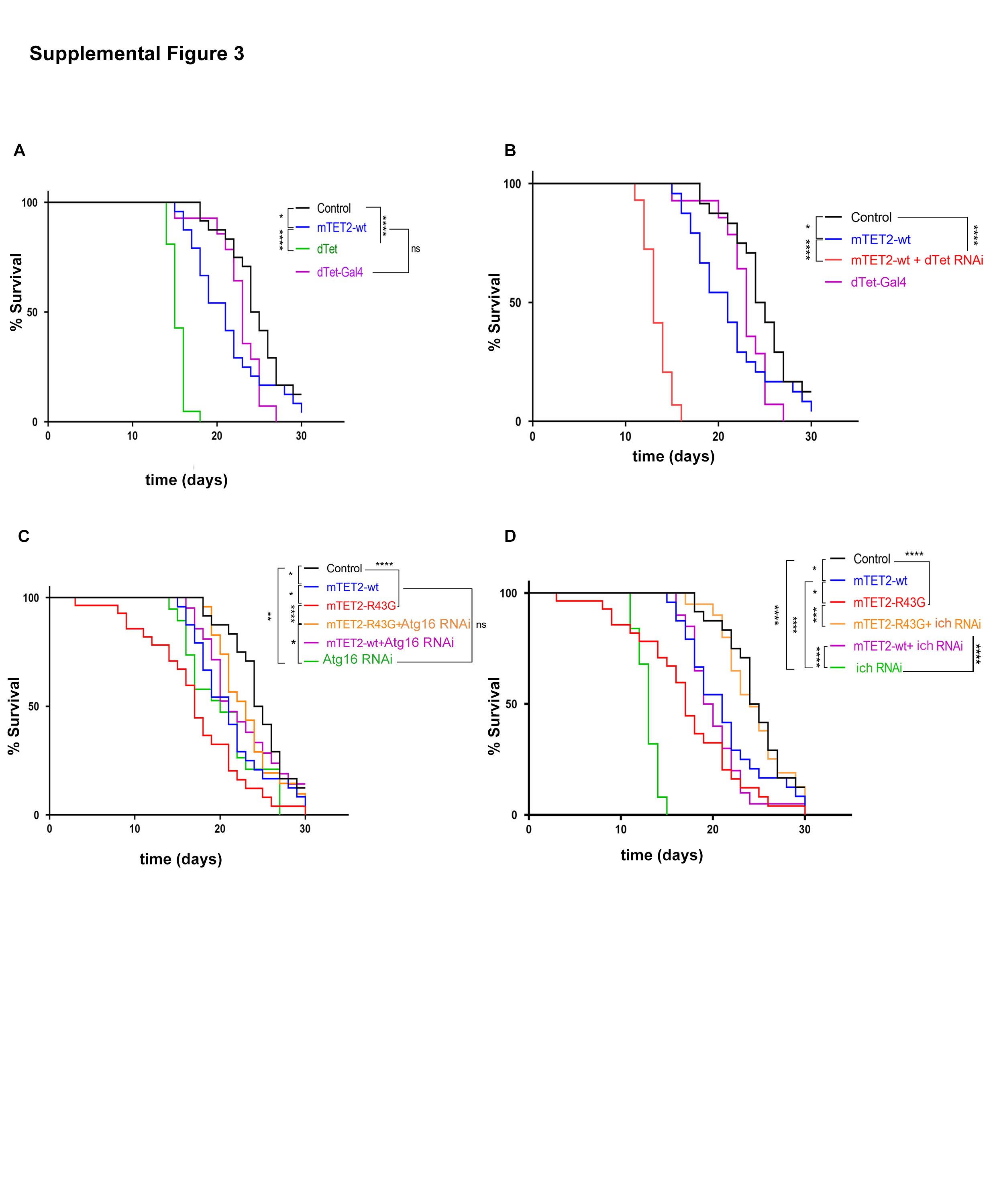

Supplement: Supplemental Material [file KEPI_A_2192375_SM8246.zip › Supplementary files/Supplemental Figure 3.tif]

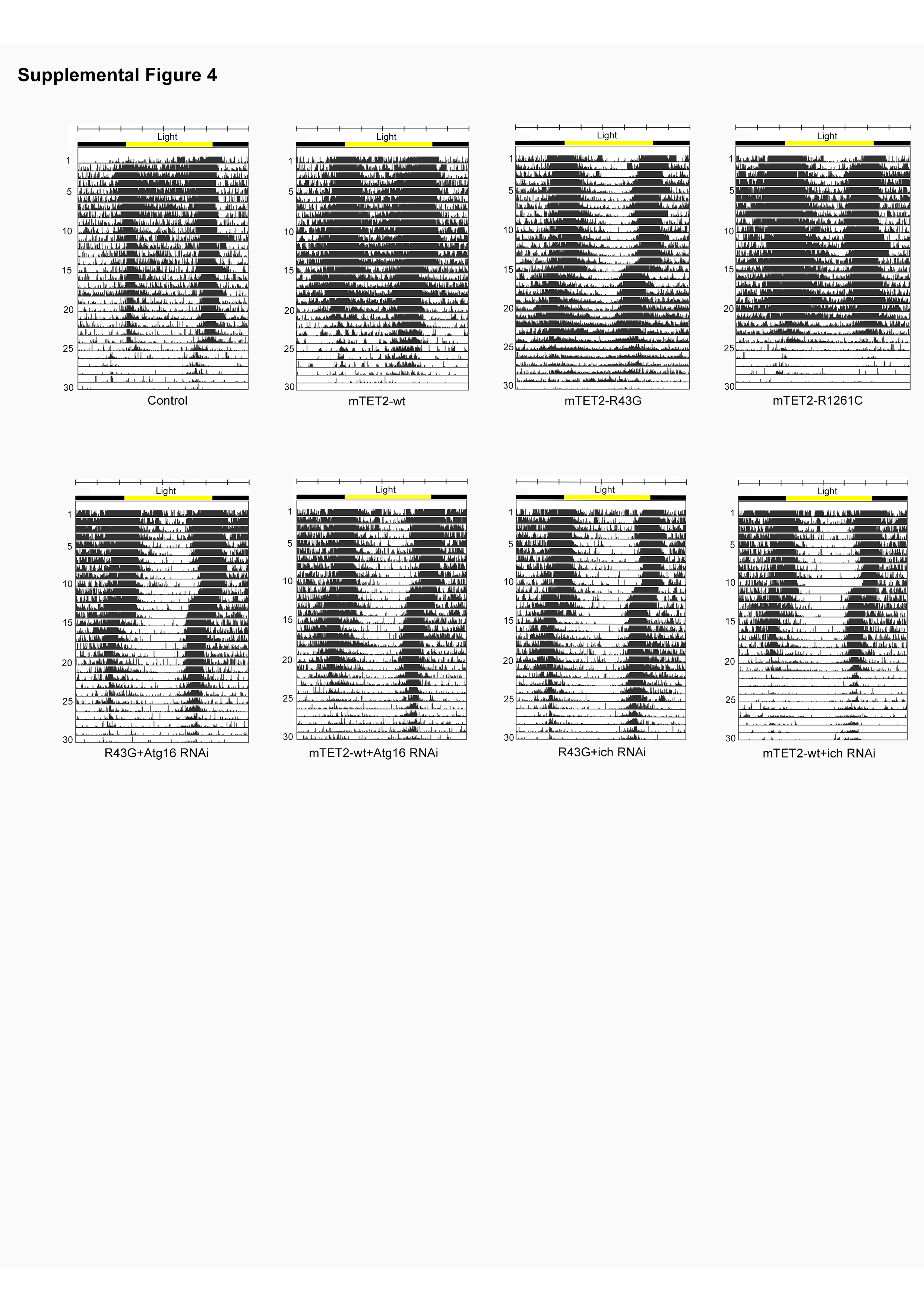

Supplement: Supplemental Material [file KEPI_A_2192375_SM8246.zip › Supplementary files/Supplemental Figure 4.tif]
